# Electrocatalytic Reduction of CO_2_ to Acetic Acid by a Molecular Manganese Corrole Complex

**DOI:** 10.1002/anie.202000601

**Published:** 2020-05-08

**Authors:** Ratnadip De, Sabrina Gonglach, Shounik Paul, Michael Haas, S. S. Sreejith, Philipp Gerschel, Ulf‐Peter Apfel, Thanh Huyen Vuong, Jabor Rabeah, Soumyajit Roy, Wolfgang Schöfberger

**Affiliations:** ^1^ Eco-Friendly Applied Materials Laboratory (EFAML) Materials Science Centre Department of Chemical Sciences Mohanpur Campus Indian Institute of Science Education and Research Kolkata 741246 West Bengal India; ^2^ Institute of Organic Chemistry Johannes Kepler University Linz Altenberger Straße 69 4040 Linz Austria; ^3^ Inorganic Chemistry I Ruhr-Universität Bochum Universitätsstraße 150 44801 Bochum Germany; ^4^ Fraunhofer UMSICHT Osterfelder Straße 3 46047 Oberhausen Germany; ^5^ Leibniz-Institut für Katalyse e. V. Albert-Einstein-Straße 29a 18059 Rostock Germany

**Keywords:** acetic acid, carbon dioxide, electrocatalysis, manganese corrole, reduction

## Abstract

The controlled electrochemical reduction of carbon dioxide to value added chemicals is an important strategy in terms of renewable energy technologies. Therefore, the development of efficient and stable catalysts in an aqueous environment is of great importance. In this context, we focused on synthesizing and studying a molecular Mn^III^‐corrole complex, which is modified on the three *meso*‐positions with polyethylene glycol moieties for direct and selective production of acetic acid from CO_2_. Electrochemical reduction of Mn^III^ leads to an electroactive Mn^II^ species, which binds CO_2_ and stabilizes the reduced intermediates. This catalyst allows to electrochemically reduce CO_2_ to acetic acid in a moderate acidic aqueous medium (pH 6) with a selectivity of 63 % and a turn over frequency (TOF) of 8.25 h^−1^, when immobilized on a carbon paper (CP) electrode. In terms of high selectivity towards acetate, we propose the formation and reduction of an oxalate type intermediate, stabilized at the Mn^III^‐corrole center.

## Graphical abstract

**Electrochemical reduction of CO_2_** to acetic acid is achieved with a molecular Mn^III^‐corrole complex. Electroreduction of Mn^III^ leads to the electroactive Mn^II^ species, which binds CO_2_ and stabilizes the reduced intermediates to allow the reduction of CO_2_ to acetic acid in a moderately acidic aqueous medium (pH 6) with a selectivity of 63 % and a turnover frequency of 8.25 h^−1^, when immobilized on a carbon paper (CP) electrode.

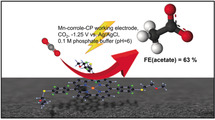

## Introduction

The growing consumption of fuels led to a drastic increase of the CO_2_ level in the atmosphere (33.1 Gt of CO_2_ in 2018^1^). A recent trend in research focuses primarily on remediation of the CO_2_ emission from non‐renewable fossil fuels as well as providing a clean, environmentally benign and cheaper alternative which can solve the current energy crisis.^2^ Getting inspired by nature, scientists found a way to mitigate this problem mainly through the use of catalysts which can mimic the role of the photosystem of green plants.^3^ In the past few decades, huge efforts have been made to solve this problem. Several classes of catalysts have been explored to reduce CO_2_ to valuable chemicals like acetic acid. Many metallic catalysts for the CO_2_ reduction reaction were investigated, like Cu,^4^ which shows a higher selectivity for the key C−C bond formation during CO_2_ reduction, where acetate was obtained with faradaic efficiencies (FE) varying from 2.5 % to 56 %.^5^ Introduction of Ag into the system, like Cu–Ag bimetallic nanoparticles^6^ marked the enhanced formation of acetate with a FE of 21.2 %. An improved FE of 60.9 % could be achieved using Fe^III^oxyhydroxide on N‐doped carbon^7^ while N‐doped nanodiamond on Si rod arrays^8^ showed a higher FE of 77.6 %. Table S1 in the Supporting Information summarizes above stated electrocatalysts for reducing CO_2_ to acetate. Aside from that, well‐designed molecular metal complexes were developed to reduce CO_2_. In this regard, some molecular Mn‐complexes like Mn(bipyridine)‐pyrene,^9^ N‐heterocyclic carbene Mn^I^ complex^10^ and other similar complexes immobilized over supports like multi‐walled‐carbon‐nanotubes (MWCNT)^11^ or Nafion membrane^12^ have been reported to reduce CO_2_ to CO. Recently, we have observed that a cobalt(III) triphenylphosphine corrole complex exhibits potential dependent CO_2_ reduction at pH 6 to produce methanol, ethanol, and low amounts of acetic acid (FE_max._ 13 % at −0.95 V vs. RHE).^13^ In this study we focus on the design of manganese corroles as potent electrocatalysts for CO_2_ reduction. Manganese(III) corroles are square‐planar complexes and initially possess no axial ligand coordination compared to the Co^III^(‐L) corroles (e.g., L=triphenylphosphine or pyridine), which renders the complex freely accessible towards axial coordination by nucleophiles (e.g., H_2_O or OH^−^).^14^, ^15^, ^16^ We have immobilized a novel manganese corrole complex (“Mn‐corrole”) on a carbon paper electrode (Mn‐Cor‐CP) to conduct heterogeneous electroreduction of CO_2_. We observed acetate as the main product in a moderate acidic aqueous medium (pH 6, phosphate buffer), at a potential of −1.25 V vs. Ag/AgCl (1 m KCl) with a FE as high as 63 % and a TOF of 8.25 h^−1^.

## Results and Discussion

### Synthesis, Fabrication and Characterization of the Electrode Material

**Figure 1:**
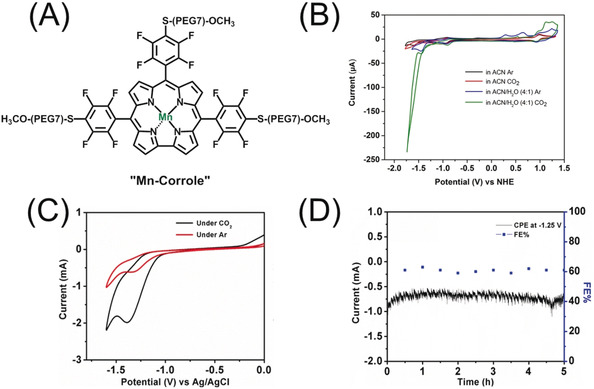
A) Chemical structure of the Mn‐corrole B) cyclic voltammetry of Mn‐corrole dissolved in ACN under Ar (black), CO_2_ (red), in ACN/H_2_O (4:1) under Ar (blue) and in ACN/H_2_O (4:1) under CO_2_ (green). C) Heterogeneous electrocatalysis of 0.5 mg cm^−2^ Mn‐corrole on carbon paper electrode under Ar (red) and CO_2_ (black) at pH 6.0 (Ag/AgCl/1 m KCl, Pt, 100 mV s^−1^). D) Controlled potential electrolysis of Mn‐corrole immobilized on a carbon paper electrode in 0.1 m phosphate buffer (pH 6) saturated with CO_2_ at −1.25 V vs. Ag/AgCl for 5 h. All homogeneous cyclic voltammetry measurements were performed with 0.1 m TBAPF_6_ as supporting electrolyte using glassy carbon as working, platinum wire as counter and a non‐aqueous pseudo‐Ag/AgCl reference electrode at a scan rate of 100 mV s^−1^.

Mn‐corrole is a molecular catalyst (Figure 1 A), which was synthesized over four steps (chemical syntheses and characterization are stated in the Supporting Information, Figure S1–S8). The free base form is the 5,10,15‐tris(2,3,5,6‐tetrafluoro‐4‐(MeO‐PEG(7))thiophenyl)corrole which complexes with Mn^III^ to form the four‐coordinated Mn(TpFPC)(‐S‐PEG(7)‐OMe)_3_ (“Mn‐corrole”). Owing to its solubility in acetonitrile and insolubility in water heterogenization over different platforms like carbon paper (CP), indium doped tin oxide (ITO) and glassy carbon (GC) leads to facile fabrication of electrodes via drop casting, which are stable in aqueous solution. This kind of functionalization of the Mn‐corrole with S‐PEG(7)‐OMe moieties leads to physisorption over a conducting surface via π–π interactions and equal distribution of the macrocycle.

XPS analysis was conducted with the catalyst immobilized on carbon paper to assess the properties of the Mn‐corrole showing the characteristic binding energy regions corresponding to Mn2p, F1s, C1s, N1s, and S2p (Figure S9A). The Mn2p scan showed two distinct peaks at 642 eV and 653 eV attributed to 2p_3/2_ and 2p_1/2_ respectively. The intensity ratio and the position of these two peaks matches well with the Mn^III^ center of previously reported Mn‐corrole.^17^ The position of the N1s band at 398.3 eV and the main peak for F1s confirms the integrity of the metal bound corrole species over the carbon paper electrode^17^, ^18^ (Figure S9A).

The XPS measurements were also performed after electrolysis where the peaks for Mn2p, C1s, N1s and S2p were positioned at the same energy regions depicting the stability of the catalyst even after the CO_2_ reduction reaction (Figure S9B). Absence of any peak at 401 eV omits the possibility of demetallation and formation of free base corrole.^18^

### Electrochemical Characterization of the Mn‐Corrole

The electrochemical properties of 0.8 mm Mn‐corrole in acetonitrile were investigated via cyclic voltammetry measurements using glassy carbon as working electrode with 0.1 m TBAPF_6_ as supporting electrolyte at a scan rate of 100 mV s^−1^. In the cathodic part, two one electron reductions at E_1/2_=−0.94 V vs. NHE (Mn^III^/Mn^II^) and E_pc_=−1.60 V vs. NHE are shown in Figure 1 B and Figure S10, where the first redox couple is reversible^14^, ^19^ whereas the second one is irreversible. Corresponding electro paramagnetic resonance (EPR) measurements, suggest the formation of a Mn^II^ radical anion at −1.60 V (Figure S11). On sweeping in the anodic region there is a metal centered and ligand centered oxidation reaction occurring. The first metal centered oxidation at E_1/2_=0.68 V and E_1/2_=0.96 V vs. NHE of Mn^III^ to Mn^IV^ is split into two waves, where the current of the first redox couple is significantly smaller than of the second one. Similar redox behavior for other triaryl‐corroles was found by Kadish and co‐workers.^14^, ^19^ This split redox‐waves is a result of two different Mn^IV^ species that can be formed, an ion pair with the anion of the electrolyte salt (Mn^IV^PF_6_) and the Mn^IV+^ cation species.^14^, ^19^ The second oxidation at E_p_=1.45 V vs. NHE is ring‐centered where Mn^IV^ is oxidized to a Mn^IV^ π radical cation.^14^ We further studied the influence of water under CO_2_ in the electrochemical system. In Figure 1 B, the comparison of Mn‐corrole dissolved in ACN (red curve) and in ACN/H_2_O=4/1 (green curve), is shown. The second reduction wave is increasing upon CO_2_ saturation at −1.43 V vs. NHE, which clearly illustrates the influence of protons in the CO_2_ reduction reaction through proton coupled electron transfer (PCET). In the cyclic voltammogram (Figure 1 B, green line) ‘‘curve crossing′′ occurs where the current on the return scan is higher than the current on the forward scan. Curve crossing stems from the increasing concentration of active catalyst (in situ generation of the active catalyst)—and so higher catalytic current—as the CV progresses.

### Heterogeneous Electrochemistry with Mn‐Corrole Towards CO_2_ Reduction

Cyclic voltammetry measurements under heterogenized conditions were conducted, to study its efficacy for the CO_2_ reduction reaction in an aqueous electrolyte solution. Mn‐corrole was physisorbed on carbon paper with an effective loading of 0.5 mg cm^−2^. Therefore, a three‐electrode electrochemical cell with Mn‐corrole‐CP as working, Ag/AgCl/1 m KCl as reference and platinum wire as counter electrode was chosen. On performing cyclic voltammetry in 0.1 m pH 6 phosphate buffer, it was found that the redox behavior of the catalyst remains almost the same as under homogeneous conditions. Cyclic voltammetry measurements were performed under Ar (red) and CO_2_ (black) with a pH 6 saturated electrolyte solution (Figure 1 C). A steep increase in current density upon CO_2_ saturation occurred, and a broad wave appeared in the cyclic voltammogram starting at −1.1 V vs. Ag/AgCl. This suggests the CO_2_ reduction is catalyzed with the immobilized Mn‐corrole on carbon paper. Further we performed controlled potential electrolysis (CPE) at varying potentials, starting from −1.1 to −1.4 V vs. Ag/AgCl over 5 h, with resulting current densities between 0.35 to 1.1 mA cm^−2^ (Figure S12 and S13). The product analysis was conducted with the resulting proton coupled nuclear magnetic resonance spectroscopy (^1^H‐NMR) and gas chromatography mass‐spectroscopy (GC‐MS) measurements (Table 1, Figure S14 and S15). Methanol and acetate are the only liquid products, CO and H_2_ are the detected gaseous compounds. CPE over 5 h with Mn‐corrole‐CP formed acetate as main product with a FE of 63 % at −1.25 V vs. Ag/AgCl and a TOF of 8.25 h^−1^ (Figure 1 D and Figure S12–S15). Additionally, the FE was measured at different intervals of time, where Figure 1 D shows that the FE remains quite constant over 5 h CPE. Methanol exhibited faradaic efficiencies between 9 and 23 %, with decreasing product formation towards lower potentials (Table 1, Figure S14A). At more negative potentials, the formation of the C_2_ product acetate is favored with a substantial increase in its FE (40 to 63 %, Table 1, Figure S14A). CO production was found only at higher potentials, −1.1 V and −1.2 V (Table 1).

**Table 1:** Comparative data for the Mn‐corrole‐CP working electrode during a 5 h electrolysis as a function of the potential in 0.1 m phosphate buffer (pH 6) saturated with CO_2_. Standard deviations were calculated after three electrochemical measurements followed by two product analysis measurements.

Potential vs. Ag/AgCl [V]	Potential vs. RHE [V]	FE % of each CO_2_ reduced products at different potential with Mn‐Cor‐CP electrode after 5 h of electrolysis								Total FE%	Charge passed (Coulomb)	
		Acetate		Methanol		CO		H_2_				
		FE%	SD	FE%	SD	FE%	SD	FE%	SD		Average	SD
−1.1	−0.524	40	4.3	23	3	31	5	–	–	94	6.3	0.2
−1.2	−0.624	55	4	20	3	20	4	–	–	95	9	0.25
−1.25	−0.674	63	3.75	16	3.5	–	–	18	4.5	97	14.4	0.3
−1.3	−0.724	60	3.55	19	2.5	–	–	19	5	98	17.1	0.5
−1.4	−0.824	61	4	9	3	–	–	25	6	95	19.8	0.8

Potential vs. RHE [V]		Overpotential η_AcOH_ [mV]	Overpotential η_MeOH_ [mV]		Partial current density [mA cm^−2^]				TON_AcOH_		TON_MeOH_	
					*j*_AcOH_		*j*_MeOH_					
−0.524		264	527		0.140		0.080		11.45		6.58	
−0.624		364	627		0.275		0.100		22.50		8.18	
−0.674		414	677		0.504		0.128		41.25		10.47	
−0.724		464	727		0.570		0.180		46.65		14.76	
−0.824		564	827		0.671		0.099		54.90		8.10	

Control measurements were performed with an unmodified CP electrode, where no product formation (methanol or acetate) was observed, which proves CO_2_ reduction ability of the Mn‐corrole (Figure S16). XPS analysis of the modified CP before and after the electrocatalysis reaction clarifies that the Mn‐corrole is stable through the CO_2_ reduction process (Figure S9). Recorded UV/Vis spectra after electrolysis of the dissolved catalyst in ACN verified that the macrocycle is still intact (Figure S17A) as well as measured heterogeneous cyclic voltammogram of the Mn‐corrole modified CP after CPE depicted no change in the electrochemical behavior of the fabricated electrode after catalysis (Figure S17B).

### Origin of the Products

**Figure 2:**
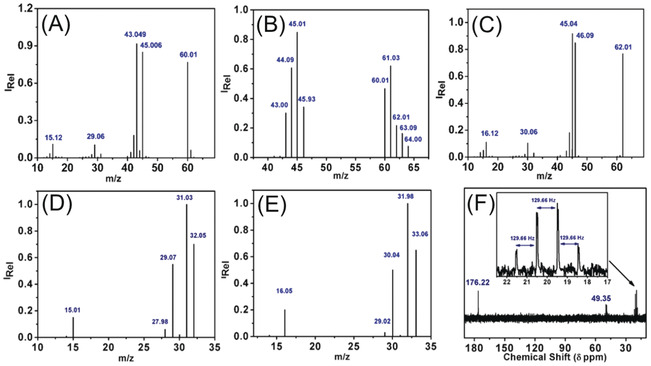
A–E) GC‐MS‐spectra of the electrolyte solution after 5 h CPE at −1.25 V vs. Ag/AgCl, pH 6, ^12^CO_2_ and ^13^CO_2_, respectively (A) of ^12^CH_3_^12^COOH and (B) deuterium substituted acetic acid, D_2_O/H_2_O (1/5) (C) ^13^CH_3_^13^COOH (D) ^12^CH_3_OH (E) ^13^CH_3_OH. F) ^1^H‐coupled‐^13^C‐NMR‐spectrum in ^13^CO_2_ saturated phosphate buffer (pH 6) after 5 h CPE at −1.25 V vs. Ag/AgCl under ^13^CO_2_ saturation.

To verify the source of protons and carbon in the products, we performed isotopic labelling experiments by using D_2_O/H_2_O confirming the proton source and ^13^CO_2_ reduction measurements for determining the carbon source. Deuterium distribution during electrolysis was investigated via CPE at −1.25 V vs. Ag/AgCl in D_2_O/H_2_O (1/5) at pH 6. The resulting GC‐MS spectrum confirmed the presence of deuterium enriched acetic acid (e.g. *m*/*z=*64), which was confirmed from the appearance of partial and fully proton–deuterium exchanged fragments (*m*/*z=*43–46) and shows a shift of *m*/*z=*1 for the respective molecular ion peak and fragments (Figure 2 A and 2B). From the resulting peaks, the one with *m*/*z=*64 can be assigned to the tetra‐deuterated (CD_3_COOD) acetic acid (Figure 2 B, S18). Among the molecular ion peak, there are also other characteristic fragments of CD_3_COOD occurring, like *m*/*z=*46 for ^.^COOD^+^ and *m*/*z=*44 for ^.^CH_2_DCO^+^ (Figure 2 B, S18). Further deuterium labelling was also observed for methanol (*m*/*z=*36, Figure S19). The D_2_O/H_2_O experiments verify that protons are incorporated via proton coupled electron transfer (PCET) in the CO_2_ reduction reaction and confirms water is the proton source in the formed products. Moreover, CPE measurements with ^13^CO_2_ were performed to confirm the carbon source. The resulting GC‐MS spectrum for acetate showed a shift of *m*/*z=*2 of the molecular ion peak (CH_3_COOH^+^, *m*/*z=*60 to 62) due to ^13^C incorporation in both carbon atoms of acetic acid (Figure 2 C). This trend is also reflected for different fragments, for example, where COOH^+^ *m*/*z=*45 shifts to *m*/*z=*46 due to ^13^C substitution. The fragment obtained at *m*/*z=*43 during the reduction of ^12^CO_2_ corresponds to ^.^CH_3_CO^+^ and shifted to *m*/*z=*45 upon replacement with ^13^CO_2_ (Figure 2 C). In the case of methanol, the molecular ion peak indicates a shift of *m*/*z=*1 upon ^13^C enrichment (*m*/*z=*32 to 33) indicating the formation of ^13^CH_3_OH (Figure 2 D and 2E). This data were further substantiated by performing ^1^H‐decoupled carbon NMR (^13^C NMR) and ^1^H NMR of the ^13^CO_2_ reduced products (Figure 2 F). The obtained ^13^C NMR spectrum reveals a quartet at 20 ppm (^1^ *J*_C,H_=129.7 Hz) and a doublet at 176.2 ppm (*J*_C,C_=55.3 Hz), which verifies the presence of fully ^13^C‐labeled acetic acid. Methanol appears at 49.3 ppm. The ^1^H NMR spectrum illustrated in Figure S14 indicates resonances at 3.2 ppm (^13^CH_3_OH, d, *J*_C,H_=134 Hz) and at 1.8 ppm (^13^CH_3_^13^COO^−^, dd, ^1^ *J*_C,H_=163 Hz, ^*2*^ *J*_C,H_=6.5 Hz). These GC‐MS, ^1^H NMR and ^13^C NMR experiments confirm that the only carbon source of methanol and acetic acid is CO_2._

From these experiments, it was confirmed that the reduced form of Mn‐corrole can catalytically activate CO_2_. Substantial increase in current density under CO_2_ atmosphere could be attributed to the adsorption and reduction of reactive CO_2_ species. The role of water was validated like mentioned above via a homogenous cyclic voltammetry measurement in a CO_2_ saturated acetonitrile solution with 20 % water content (Figure 1 B, green curve). There was a characteristic increase in the second Mn reduction current observed, which suggests the formation of the catalytically active species with subsequent CO_2_ reduction reaction. This signifies the need for an intervening pathway of PCET in the presence of a proton donor to facilitate CO_2_ electroreduction.

### Plausible Mechanistic Pathway for CO_2_ Reduction

**Figure 3:**
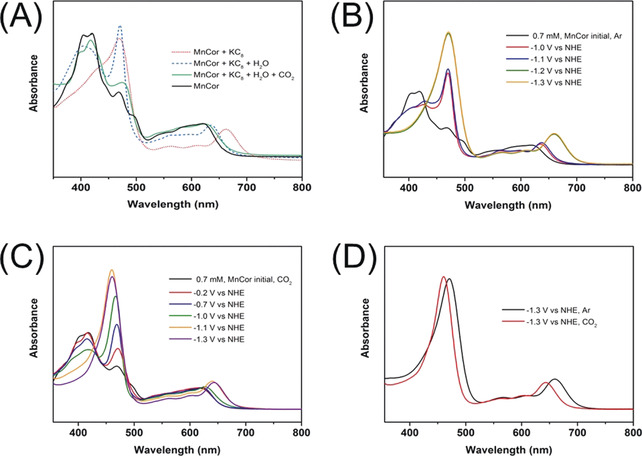
A) EAS of Mn‐corrole in acetonitrile under argon (black solid line), the chemically reduced form in presence of KC_8_ (red dotted line), after the addition of water (blue, dashed line), and subsequent dosage of CO_2_ (green solid line). B–D) Potential dependent SEC‐UV/Vis of 0.7 mm Mn‐corrole in acetonitrile with 2 % water and 0.2 m TBAPF_6_ as electrolyte after 2 min CPE (B) under Argon (C) under CO_2_, and (D) comparison of UV/Vis spectra observed at −1.3 V vs. NHE under Argon (black) and CO_2_ dosage (red). SEC‐UV/Vis measurements were recorded with a light transparent platinum mini‐grid as working, as counter and an Ag‐microwire as pseudo‐reference electrode.

To have a deeper insight into the electron transfer phenomena obtained from the cyclic voltammetry investigations we performed chemical reduction of the Mn^III^‐corrole in acetonitrile as solvent. To demonstrate the charge transfer occurring between Mn‐corrole and CO_2_, we have reduced Mn^III^‐corrole typically by the addition of KC_8_ in dry acetonitrile solution. The electronic absorption spectrum (EAS) encounters a prominent change to a single Soret‐band maximum at 470 nm with Q‐bands at 561 nm, 610 nm, and 663 nm (Figure 3, red dashed line). After the addition of water, the Soret band exhibits splitting with absorption maxima at 406 nm and 471 nm, which indicates the formation of MnCor(OH)_2_ species (Figure 3, blue dashed line). This species was first investigated by Kadish and co‐workers.^14^, ^15^ After exposing the solution to CO_2_, we observe a distinct change in the EAS spectrum (Figure 3, green solid line). The obtained UV/Vis spectrum shows striking similarity to the one observed for Mn^III^‐corrole (Figure 3, black line). The position and intensity of the band at 475 nm is slightly altered compared to the initial spectrum for the Mn‐corrole, which is attributed to the axial ligation of CO_2_ at the Mn site. During the subsequent PCET the formation of Mn^III^‐CO_2_H is facilitated. At this step, the reaction can take different routes one of which is another PCET on carbon site to form HCOOH, which can eventually lead to formation of other C_1_ products like CH_3_OH (Figure S20). On the other hand, second protonation on the hydroxyl oxygen followed by dehydration generates CO. Another possibility is the C−C bond formation to generate an oxalate type intermediate. From here, successive reduction of the oxalate leads to the formation of acetate as the final product (Figure S21).

**Scheme 1:**
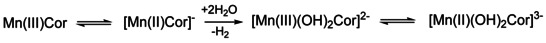
Redox reactions of Mn‐corrole in ACN with 2 % water.^14^, ^15^

Furthermore, the electrochemical redox behavior of Mn^III^‐corrole was characterized under argon and CO_2_ atmosphere via spectroelectrochemical UV/Vis (SEC‐UV/Vis) measurements in an optical transmission thin layer electrode cell (OTTLE cell, see experimental section in the Supporting Information). Therefore, a light transparent platinum mini‐grid acts as working, as counter and Ag‐microwire as pseudo‐reference electrode. Measurements were pretreated for 1 min at constant potential, afterwards the UV/Vis recording was started with additional electrolysis of 1 min at the same potential. The first one electron reduction at −1.0 V vs. NHE in ACN with 2 % water under Ar ascribes the electrochemical reduction of initial Mn^III^ to Mn^II^ with subsequent chemical reaction to [Mn^III^(OH)_2_]^2−[14, 15]^ (Scheme 1 and Figure 3 B, red line).

This electrogenerated species shows a splitted Soret band at 428 nm, a significant intense band at 470 nm and Q‐bands at 563, 597 and 637 nm, which is similar to the formed bis‐hydroxy species after chemical reduction and water addition. When moving on to more negative potentials like ‐1.3 V vs. NHE, [Mn^III^(OH)_2_]^2−^ is reduced to [Mn^II^(OH)_2_]^3−^, where one sharp visible band at 471 nm, bathochromic shifted Q‐bands at 567 nm, 612 nm and one intense Q‐band at 660 nm was formed (Figure 3 B green and orange line).^14^, ^15^ On performing measurements in ACN with 2 % water under CO_2_ at various negative potentials (−0.2 to −1.3 V), a decrease of the initial Soret‐band at 417 nm with a consequently increase of a hypsochromic shifted visible band from 470 nm to 461 nm and an intense Q‐band at 643 nm were observed (Figure 3 C). At the first reduction potential (−1.0 V) of Mn^III^‐corrole, the visible band at 467 nm (Figure 3 C, green line) was more intense compared to that under Ar (470 nm), which can be ascribed to the additional axial ligation of CO_2_ to the Mn‐site. On shifting the applied potential to −1.3 V vs. NHE, one bathochromic shifted sharp visible band was formed at 461 nm (Figure 3 D, red line) compared to 471 nm under Ar (Figure 3 D, black line). This suggests, that [Mn^II^(OH)_2_]^−^ is the possible catalytic active species, where CO_2_ can coordinate to the metal site to be transformed via PCET processes into respective reaction intermediates and products.

**Figure 4:**
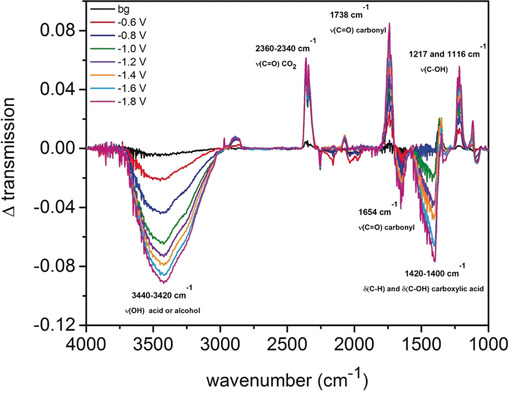
SEC‐FTIR of 15 mm Mn‐corrole in ACN/H_2_O (4/1) and 0.1 m TBAPF_6_ under CO_2_ at various potentials from −0.6 to −1.8 V vs. NHE. Glassy carbon was used as working, platinium wire as counter and silver wire as pseudo‐reference electrode. (bg=background).

For further insights in possible reaction intermediates we performed spectroelectrochemical attenuated total reflection Fourier‐transform infrared spectroscopy measurements (SEC‐ATR‐FTIR) at various potentials from −0.6 to −1.8 V vs. NHE. Figure 4 illustrates increasing IR‐bands at 3440–3420 cm^−1^ (O−H vibrational stretching), 1654 cm^−1^ (C=O vibrational stretching) and 1420–1400 cm^−1^ (C−H and C−OH stretching), which suggests the stepwise formation of an alcohol or acid. The respective IR‐band for CO_2_ at 2360–2340 cm^−1^ is decreasing towards lower potentials, verifying that CO_2_ is converted. Further decreasing IR‐bands at 1738 cm^−1^ (C=O vibrational stretching), 1217 cm^−1^ (vibrational stretching C−OH) and 1116 cm^−1^ (vibrational stretching C−OH) suggest the formation and consumption of the proposed Mn−C(H)OOH intermediate to further products, like methanol or acetic acid.

Mechanistic investigations show that metal and ligand centered activity of porphyrins^20^ or phthalocyanines^21^ are a determining factor for the selectivity of CO and HCOO^−^/HCOOH formation. On similar trails, Mn‐corrole acting as metal active corrole species possesses the ability to stabilize a metal bound carboxyl group. Due to this, CO_2_ reduction reaction with Mn‐corrole preferably results in the formation of the 2 e− reduced CO, 6 e− reduced methanol or via 8 e− reduced acetate (Supporting Information p. 29, Equation S1–S14). Presence of protons (pH 6) lowers the activation energy for the first PCET towards the O‐atom^22^ of CO_2_ resulting in the formation of a carboxyhydroxyl intermediate stabilized on Mn^III^‐corrole site. This stabilization results not only in achieving a low energy cost but also plays decisive role in the selectivity of the products. From this step another PCET on the C‐atom leads to the formation of HCOOH type intermediate which on subsequent reduction forms methanol. On the other hand a PCET over the O‐atom of the carboxyhydroxyl intermediate resulted in the formation of CO. Higher selectivity for acetate achieved in this work, originated from the Lewis acidity of the Mn^III^ center, which has a higher tendency to bind with the Lewis basic O‐site of the carboxyl group, hence facilitating the C−C dimerization leading to an oxalate type intermediate.^8^, ^23^ This proposition was further substantiated by DFT calculations (Figure S22–S25), which showed a potential stabilization of −1.04 eV upon C−C dimerization. From this step, consecutive reduction results in the formation of acetate (Supporting Information p. 29, Equation S1–S14). During CO_2_ electrolysis over Mn‐corrole‐CP electrodes no trace of oxalate or formate was detected. To get a better idea of the mechanistic pathways we performed electrochemical reduction of possible reaction intermediates. The reduction of 10 mm formic acid by Mn‐corrole under similar electrocatalytic conditions generates methanol as the only liquid product (Figure S20) with no gaseous components. From these observations we come to the conclusion that the reduction of formic acid only yields methanol, thus it serves as an important 2 e− reduced intermediate in CO_2_ reduction to methanol. When reduction of 10 mm oxalate in pH 6 phosphate buffer was performed with Mn‐corrole‐CP as cathode, acetate (Figure S21) was obtained exclusively which supports our proposition for the formation of an oxalic acid type intermediate which follows multiple reduction steps on the pathway of acetate formation (Supporting Information p. 29, Equation S1–S14).

## Conclusion

To conclude, in this work we have explored a molecular manganese‐corrole‐CP catalyst for the electrochemical CO_2_ reduction to produce acetate at −1.25 V vs. Ag/AgCl over 5 h electrolysis time with a FE of 63 %. Herein the S‐PEG(7)OMe modified A_3_‐corrole is designed in such a way that it exhibited the prerequisite 8 e− reduction of CO_2_ in a selective manner. We suggest a mechanistic pathway which involves the formation and reduction of a Mn^III^ bound oxalate intermediate to acetate which was further substantiated by reduction studies of oxalic acid and DFT calculations. This work opens up opportunities for direct production of acetic acid from CO_2_ with high selectivity, which currently involves a multi‐step process via production of methanol and its carbonylation starting from the synthesis of syngas. In summary, we presented an efficient approach for direct acetic acid formation via electrocatalytic CO_2_ reduction using a Mn‐corrole based molecular catalyst. Further studies on this kind of catalysts will help in designing more superior molecular catalysts for the purpose of storing energy through chemical bond formation in a selective manner.

## Supporting information

As a service to our authors and readers, this journal provides supporting information supplied by the authors. Such materials are peer reviewed and may be re‐organized for online delivery, but are not copy‐edited or typeset. Technical support issues arising from supporting information (other than missing files) should be addressed to the authors.

Supplementary
