# Supplementary material for: Electrocatalytic Reduction of CO2 to Acetic Acid by a Molecular Manganese Corrole Complex
Source: Angew Chem Int Ed Engl. 2020 May 8;59(26):10527–34. doi: 10.1002/anie.202000601 (PMC7540269; doi:10.1002/anie.202000601)
Supplement: Supplementary file 1 — Supplementary [file ANIE-59-10527-s001.pdf]

Angewandte Chemie
Eine Zeitschrift der Gesellschaft Deutscher Chemiker

Supporting Information

Electrocatalytic Reduction of CO_2 to Acetic Acid by a Molecular Manganese Corrole Complex

Ratnadip De[+], Sabrina Gonglach[+], Shounik Paul[+], Michael Haas, S. S. Sreejith, Philipp Gerschel, Ulf-Peter Apfel, Thanh Huyen Vuong, Jabor Rabeah, Soumyajit Roy, and Wolfgang Schöfberger**

anie_202000601_sm_miscellaneous_information.pdf

Supporting Information

Content

Benchmarking 2
Experimental Section 3
Characterization of the free base- and Mn-Corrole 8
Characterization of Mn-Corrole on Carbon Paper electrode 12
Electrochemical measurements 14
Calculation of Faradaic efficiency and Turn over frequency 18
After catalysis characterization 21
Effect of Deuterium substitution 23
Reduction studies of possible intermediates 24
DFT calculations 26
References 33

Benchmarking

Table S1: Benchmarking of well-known electrocatalysts reducing CO_2 to acetate.

entry	Catalyst	Potential (V) /	Current density (mA cm^{-2})	FE (%) for Acetate	Other Products	Electrolyte	Reference
1	Cu Foil	-1.05 V vs RHE	-7	0.3	C_2H_4, CH_4	0.1 M $KHCO_3$	[1]
2	Nanostructured iron (III) oxyhydroxide on nitrogen-doped carbon	-0.5 V vs Ag/AgCl (3 M)	-0.36	60.9	HCOOH	0.05 M $KHCO_3$	[2]
3	Mesostructured Cu	-0.57 V vs RHE	-0.66	2.54	CH_4, C_2H_6, C_2H_4, *n*-C_3H_7OH	0.1 M $KHCO_3$	[3]
4	N-doped nanodiamond/ Si rod array	-1.0 V vs RHE	-1.0 (approx)	77.6	no other products	0.5 M $NaHCO_3$	[4]
5	Cu-Ag poly	−0.72 V vs. RHE	-78.8	4.2	CH_4, C_2H_4, C_2H_5OH	1 M KOH	[5]
6	Cu_2Ag_3	-1.33 V vs RHE	-0.85	21.2	CO, CH_4, HCOOH	0.5 M $KHCO_3$	[6]
7	Cu NP/CNT	-1.4 V vs Ag/AgCl	-100	56	HCOOH, CH_3OH	0.5 M $KHCO_3$	[7]
8	Mn-Cor-CP	-1.25 V vs Ag/AgCl	-0.8	63	CH_3OH, CO	0.1 M phosphate buffer (pH 6)	This work

Experimental Section

Materials

All solvents and chemicals were purchased from commercial suppliers and were used without further purification, unless otherwise noted. The aqueous solutions were prepared using Merck HPLC grade water. Dry acetonitrile stored within a molar sieve MB-SPS-7, M. Braun Inert gas-System GmbH (Garching, Germany) under argon and HPLC grade water has been used for all spectroscopic and electrochemical measurements. Tetrabutylammonium hexafluorophosphate ($TBAPF_6$, Alfa Aesar) was recrystallized twice, with absolute ethanol, dried under high vacuum and stored under argon before use. 5,1015-tris(pentafluorophenyl)corrole was synthesized according to literature[8].Toray Carbon Paper, 19x19 cm, was purchased from Alfa aesar, D_2O (D, 99.96 %) used in NMR and isotopic study, was purchased from Cambridge Isotopic Laboratory. $^{13}CO_2$ (99.0 atom %) from Icon isotopes U.S.A, was used for isotope labelling study. H_2O^{18} (98.0 atom %) was purchased from Aladdin Industrial Corporation. CO_2 and Ar used in electrochemical measurements were purchased from Praxair with a purity of 99.95 %.

Material synthesis and characterization

Moisture sensitive syntheses were carried out under argon atmosphere using normal Schlenck-technique and under reduced light conditions.

Free base 5,10,15-tris(2,3,5,6-tetrafluoro-4-(MeO-PEG(7)thiophenyl)corrole H_3TpFPC(-S-PEG(7)-OMe)$_3$[9]

29 mg sodium hydride (95 %, 19 equiv., 1.19 mmol) were dissolved in 5 mL THF_{dry} in a 25 mL Schleck-flask under argon atmosphere. Further 69.4 µL MeO-PEG(7)-thiol (3.2 equiv., 74 mg, 0.207 mol) were added with subsequent addition of 50 mg 5,10,15-trispentafluorophenylcorrole (H_3TpFPC) (0.0632 mol) in 5 mL $THF_{dry,}$ and the reaction was stirred at room temperature for 20 min. The reaction was monitored *via* TLC (aluminium oxide 60, neutral) and mass spectroscopy. After complete conversion, 10 mL water were added to quench the reaction. 10 mL 0.1 M HCl were added to the water-THF phase and extracted three times with 20 mL ethylacetate. The combined organic phases were dried over Na_2SO_4, filtered and evaporated to dryness. Purification was performed *via* column chromatography (aluminium oxide 60, basic, activity level II, DCM:MeOH (200:1). H_3TpFPC(-S-PEG(7)-OMe)$_3$ (102 mg, 0.0565 mol, 90 %) was obtained as a turquoise-purple solid.

R_f = 0.31 (DCM/MeOH 100:1); ^{1}H-NMR (300.1 MHz, $CDCl_3$, 25 °C): δ = 9.09 (d, $^3J_{H,H}$ = 4.14 Hz, 2H; pyrrole-H), 8.80 (d, $^3J_{H,H}$ = 4.38 Hz, 2H; pyrrole-H), 8.61 – 8.58 (m, 4H; pyrrole-H), 3.91 (t, $^3J_{H,H}$ = 6.20 Hz, 6H; CH_2), 3.74 – 3.71 (m, 12H; CH_2), 3.68-3.65 (m, 12H; CH_2), 3.61 (bs, 12H; CH_2), 3.56-3.55 (m, 18H; CH_2), 3.52-3.49 (m, 6H; CH_2), 3.42-3.39 (m, 12H;

CH_2), 3.26 (s, 9H; CH_3) ppm; ^{19}F-NMR (282.4 MHz; $CDCl_3$; 25 °C): δ = -133.63 (dd, $^3J_{F,F}$ = 25.20 Hz, $^4J_{F,F}$ = 11.87 Hz, 4F; F_{ortho}), -134.06 (dd, $^3J_{F,F}$ = 25.76 Hz, $^3J_{F,F}$ = 12.12 Hz, 2F; F_{ortho}), -137.56 (dd, $^3J_{F,F}$ = 25.50 Hz, $^4J_{F,F}$ = 12.21 Hz, 2F; F_{meta}), -138.22 (dd, $^3J_{F,F}$ = 24.94 Hz, $^4J_{F,F}$ = 11.73 Hz, 4F; F_{meta}) ppm; ^{13}C-NMR (125.8 MHz, $CDCl_3$, 25 °C): δ =147.3 (d, $^1J_{C,F}$ = 245.7 Hz; Ar.-CF), 147.1 (d, $^1J_{C,F}$ = 246.5 Hz; Ar.-CF), 145.7 (d, $^1J_{C,F}$ = 249.6 Hz; Ar.-CF), 145.3 (d, $^1J_{C,F}$ = 250.8 Hz; Ar.-CF), 147.0 (d, $^1J_{C,F}$ = 247.2 Hz; Ar.-CF), 146.9 (d, $^1J_{C,F}$ = 247.4 Hz; Ar.-CF), 146.3 (d, $^1J_{C,F}$ = 247.2 Hz; Ar.-CF), 146.2 (d, $^1J_{C,F}$ = 247.4 Hz; Ar.-CF), 142.7-140.0 (m; CC), 134.4 (s; CC), 130.4 (s; CH), 127.6 (s; CH), 126.3 (s; CH), 122.0-121.1 (m; CH), 120.9-120.5 (m; CH), 118.5-118.2 (m; CH), 117.0 (s; CH), 115.9-115.3 (m; CH), 99.1 (s; CC), 95.4 (s; CC), 71.8 (s; CH_2), 70.7-70.4 (m; CH_2), 58.9 (s; CH_2), 34.4 (s; CH_3) ppm; HRMS (ESI): *m/z* calcd for $C_{82}H_{104}F_{12}N_4O_{21}S_3$: 1805.6236 $[M+H]^+$, 1827.6061 $[M+Na]^+$; found: 1805.6273 $[M+H]^+$, 1827.6096 $[M+Na]^+$; UV/Vis (ACN): λ_{max} (log($\mathcal{E}$)): 415 (5.2), 568 (4.4), 606 (4.2) nm.

Mn-Corrole: Mn (TpFPC)(-S-PEG(7)-OMe)$_3$

212 mg H_3TpFPC(-S-PEG(7)-OMe)$_3$ (0.1174 mol) and 320.5 mg manganese(II)acetate tetrahydrate (1.3077 mol) were dissolved in 8 mL DMF_{dry} in a 25 mL two-neck round bottom-flask under argon atmosphere. The reaction mixture was heated to reflux for 30 min and the reaction progress was monitored *via* UV/Vis. After complete metalation, the mixture was extracted five times with ethylacetate and an excess of a saturated sodium chloride solution. The organic phase was washed with water, dried over sodium sulfate, filtered and evaporated under reduced pressure. Purification was done *via* column chromatography (aluminium oxide 60, basic, activity level II, DCM:MeOH (200:1), where the green product Mn (TpFPC)(-S-PEG(7)-OMe)$_3$ was obtained with 87 % yield (110 mg, 0.0592 mmol).

R_f = 0.47 (DCM/MeOH 100:1); ^{19}F-NMR (282.4 MHz, $CDCl_3$, 25 °C): δ = -115.52 (bs; 3F), -130.47 (s; 9F); HRMS (ESI): *m/z* calcd for $C_{82}H_{101}F_{12}MnN_4O_{21}S_3$: 1857.5382 $[M+H]^+$, 1874.5653 $[M+NH_4]^+$; found: 1857.5373 $[M+H]^+$, 1874.5601 $[M+NH_4]^+$; UV/Vis (ACN): λ_{max} (log($\mathcal{E}$)): 375 (4.6), 404 (4.8), 419 (4.8), 443 (4.5), 471 (4.5), 496 (4.3), 547 (4.1), 583 (4.2), 620 (4.2) nm.

Methods

Preparation of the working electrode

The carbon paper was immobilized with the Mn-Corrole to obtain the working electrode (A=1 cm^2). Therefore, a 1 mM solution of Mn-Corrole in acetonitrile was drop casted over the carbon paper to achieve a loading of 0.5 mg cm^{-2}. Further the prepared electrodes were washed with high purity water (18 MΩ) to remove the excess of acetonitrile completely and were dried under high-vacuum. These electrodes were used as working electrode for all heterogeneous electrochemical measurements.

Cyclic Voltammetry (CV) and controlled potential electrolysis (CPE)

All air and water sensitive measurements were conducted under argon-atmosphere and with dry solvents. The electrochemical measurements have been performed in CHI 400A or Pine WaveDriver 20 DC Bipotentistat/Galvanostat workstation. A three-neck gas tight cell has been used for all electrochemical experiments. Every time before performing controlled potential electrolysis the working electrodes were cleaned thoroughly using the electrolyte. Pt wire as counter electrode and Ag/AgCl (1M KCl) as reference electrode were taken in all cases. Electrolysis was carried out in 0.1 M phosphate buffer with pH 6, if not mentioned otherwise. For electrochemical measurements conducted in non-aqueous medium, a 0.8 mM Mn-Corrole solution in acetonitrile with $TBAPF_6$ (0.1 M) as the supporting electrolyte was prepared and a three electrode setup with glassy carbon as working, platinum wire as counter and a nonaqueous pseudo-Ag/AgCl referecene electrode were used. Ferrocen was added at the end of nonaqueous measurements as an internal standard. The converted potentials against Fc/Fc^+ were converted to V vs NHE with the conversion of +0.630 V vs NHE according to literature[10]. Controlled potential experiments for CO_2 reduction were done under CO_2 saturated pH 6 phosphate buffer with continuous CO_2 purging, while that for water oxidation was performed with pH 6 phosphate buffer.

Spectroelectrochemical-ultraviolet-visible experiments (SEC-UV-vis)

SEC-UV-vis measurements were performed with an Omni Specac optical transmission thin layer electrode cell (OTTLE cell, optical path less than 0.2 mm) with CaF_2 polished crystal optic windows (41mm x 23mm x 6mm) from Apollo Scientific[11]. The cell consists of a light transparent platinum mini grid (32 wires per cm^2) as working, a platinum mini grid as counter and a silver wire as pseudo-reference electrode. Potentials were applied with an Emstat[3] potentiostat electrochemistry station. Time resolved UV-vis spectra were recorded with a Varian CARY 100 Bio spectrometer. The potential was held for 1 min before the UV-vis spectra were recorded. The UV-vis measurment was completed after 1 min.

Spectroelectrochemical fourier-transform infrared spectroscopy (FTIR) measurements (SEC-FTIR)

Infrared spectroelectrochemistry (SEC-IR) measurements were carried out on a SP-02 cell (Spectroelectrochemistry Partners) attached to a Bruker Tensor 27 FT-IR spectrometer with a Pike Miracle ATR unit. A PalmSens4 was used as potentiostat with a standard three-electrode setup (WE: glassy carbon electrode, RE: Ag wire, CE: Pt wire). All measurements were carried out in CO_2 saturated acetonitrile/water (4:1) mixtures containing 15 mM of the "Mn-Corrole" species and 0.1 M $TBAPF_6$ as electrolyte. Prior to each experiment, the electrochemical cell was degassed with Ar for 10 min and the atmosphere was maintained throughout the measurement. Furthermore, the working electrode was prepared by successive polishing with 1.0 and 0.3 µm sandpaper and subsequent sonication in acetonitrile for 10 minutes. The WE was placed 200 µm above the ATR crystal and the potential was held for 800 s before the IR scan was initiated. The IR measurement (128 scans) was completed in roughly 260 s.

Electro paramagnetic resonance spectroscopy (EPR)

Electro paramagnetic resonance spectroscopic measurements were performed on a Bruker EMX CW-micro X-band EPR spectrometer equipped with an ER4119HS high-sensitivity resonator, with a microwave power of Ca 6.9 mW and modulation frequency and amplitude of 100 kHz and 5 G, respectively. The EPR spectrometer was equipped with a temperature controller and liquid N_2 cryostat for low temperature measurements.

For electrochemical reduction, 1 mM Mn-Corrole solution in acetonitrile with $TBAPF_6$ (0.1 M) as the supporting electrolyte, was prepared in the EPR-tube, Ag/AgCl (0.1 M KCl) as the reference and Pt wire as the counter electrode were used. A Statron was used as potentiostat to control the potential for electrochemical reduction. The EPR spectra were recorded for the fresh "Mn-Corrole" and immediately after electrochemical reduction at 110 K.

Product analysis by GC-MS

GC-MS analysis was performed using Trace 1300 GC and ISQ QD single quadrapole GC-MS system equipped with a DB-624 capillary column (30 m × 0.32 mm × 0.2 µm). On the other hand gaseous products of the electrolyte were analyzed by a TCD detector equipped with a CarboPLOT 007 capillary column (25 m × 0.53 mm × 0.25 µm). For the detection of acetate the electrolyte was first acidified by $HCl_{conc.}$ and then the acetic acid was extracted into DCM. This extracted solution was then used for GC-MS analysis. Methanol was analysed by heating the electrolyte solution in a head space vial at 60 ^{0}C for 10 minutes and taking the 100 µL head space gas for the analysis. During the electrolysis in closed cell, 200 µL of the head space gas was taken for the quantification of evolved gas. CO and H_2 were detected during the electrolysis in the cathodic region. For the quantification purpose the peak area was converted into gas volume using calibration curve.

Product detection and quantification by ^{1}H-NMR

All liquids products have been quantified by a JEOL ECS-400 NMR spectrometer using D_2O as solvent. 50 µL of 25 mM phenol and 7 mM DMSO solution was used as internal standard. For this purpose we have followed a previously reported procedure.[1] 400 µL analyte was taken directly from the reaction mixture for the ^{1}H-NMR analysis. Further suppression of water peak was conducted in order to make the CO_2 reduced product peaks visible. Acetate along with methanol were found as only reduced product from ^{1}H-NMR with corresponding peaks at 1.79 ppm and 3.23 ppm respectively (Figure S14B and S15). Number of scans and other spectral acquisition parameters were kept fixed during all acquisitions. During the quantification of the samples, each peak was normalized with respect to the DMSO peak at 2.6 ppm.

^{13}C-NMR analysis was done using a Bruker (^{1}H 500 MHz, ^{13}C 126 MHz) NMR spectrometer. For ^{13}C enriched acetate and methanol, proton coupled ^{13}C-NMR measurements were performed.

Electronic Absorption Spectroscopy (EAS)

For recording UV-vis spectra, a 0.02 mg mL^{-1} Mn-Corrole in acetonitrile solution was prepared as well as for chemical reduction experiments. EAS was performed using a Shimadzu UV-vis spectrophotometer (UV 1800) in UV-Probe 2.5 or a Varian CARY 100 Bio spectrometer. After controlled potential electrolysis in cathodic and anodic regions, the Mn-Corrole was re-dissolved from the working electrode to check its stability after the electrolysis.

High resolution mass spectroscopy (HRMS)

High resolution mass spectra were recorded with a Agilent 6520 Q-TOF mass spectrometer with an ESI source and an Agilent G1607A coaxial sprayer. The Analyses were conducted in the positive and negative ionization mode.

X-ray photoelectron spectroscopy measurements (XPS)

XPS was performed by using a Theta Probe, Thermo Scientific, UK, using monochromatic Cu Kα X-rays (hν = 1486.6 eV), spot size 400 microns and with a photoelectron take-off angle of 90° with respect to the surface plane. Charge neutralization was achieved using dual flood gun. The binding energies were corrected using the C1s peak at BE = 284.6 eV that arises from adventitious hydrocarbon**.** Survey spectra were recorded with a pass energy of 200 eV with energy steps of BE 1 eV, the detailed spectra were recorded with a pass energy of 50 eV and with energy steps of BE 0.05 eV.

Characterization of the free base- and Mn-Corrole

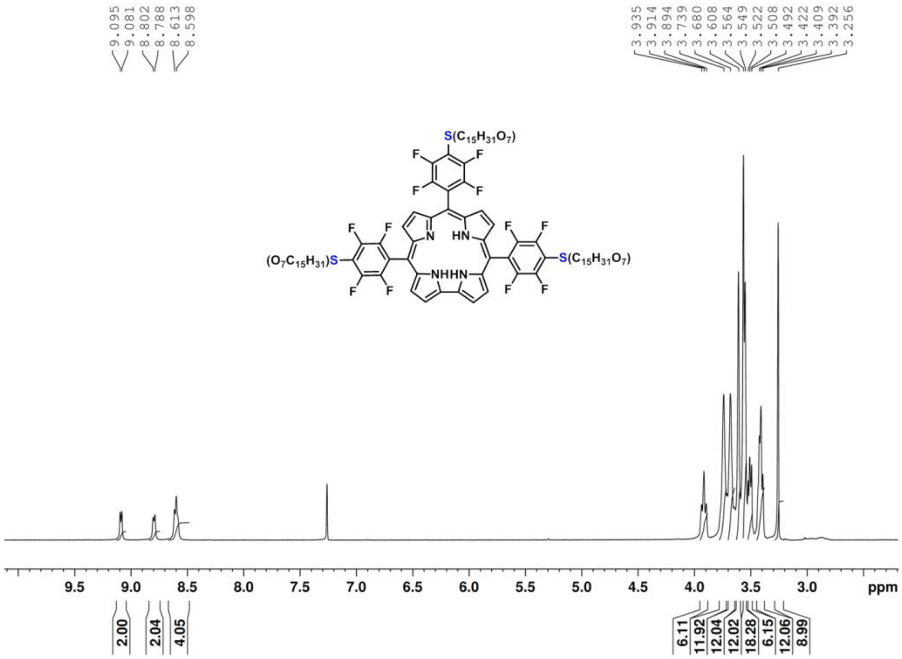

Figure S1: ^{1}H-NMR of 5,10,15-tris(2,3,5,6-tetrafluoro-4-(MeO-PEG(7)thiophenyl)corrole H_3TpFPC(-S-PEG(7)-OMe)$_3$ in $CDCl_3$.

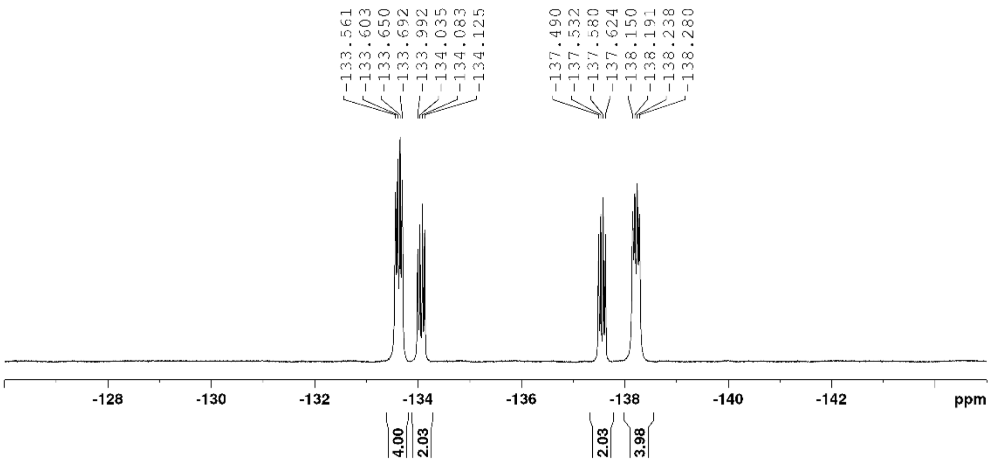

Figure S2: ^{19}F-NMR of 5,10,15-tris(2,3,5,6-tetrafluoro-4-(MeO-PEG(7)thiophenyl)corrole H_3TpFPC(-S-PEG(7)-OMe)$_3$ in $CDCl_3$.

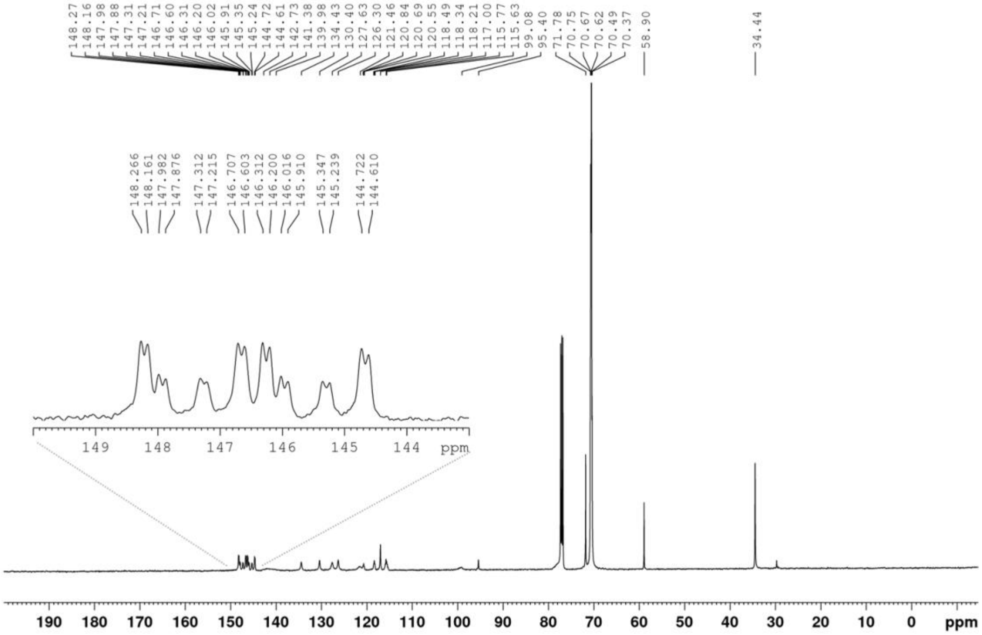

Figure S3: ^{13}C-NMR of 5,10,15-tris(2,3,5,6-tetrafluoro-4-(MeO-PEG(7)thiophenyl)corrole H_3TpFPC(-S-PEG(7)-OMe)$_3$ in $CDCl_3$.

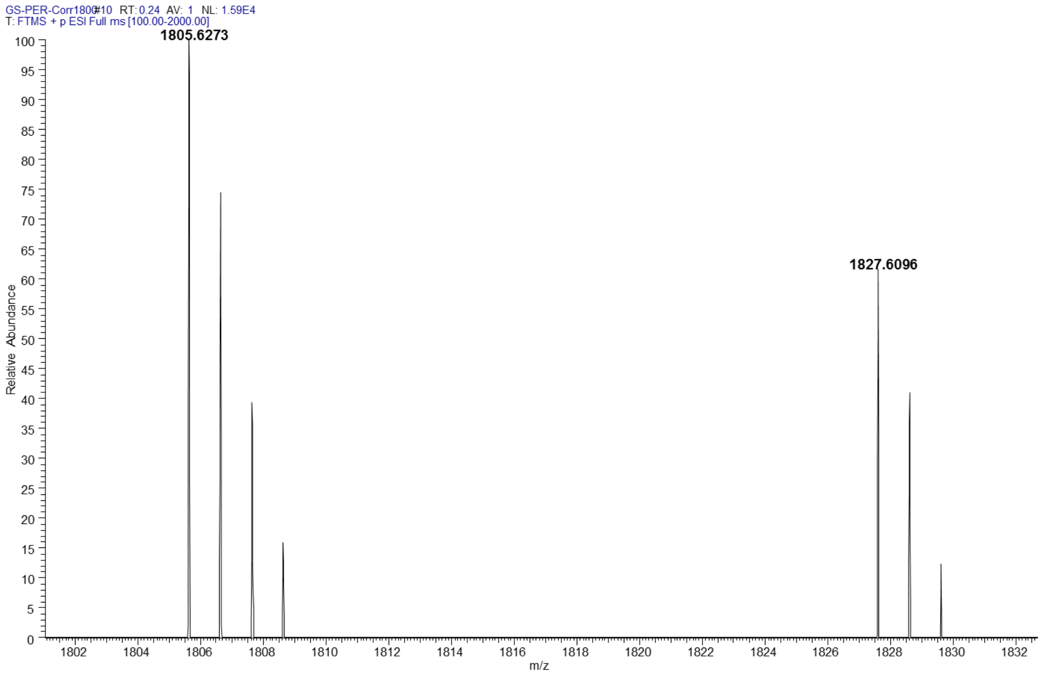

Figure S4: HRMS of (2,3,5,6-tetrafluoro-4-(MeO-PEG(7)thiophenyl)corrole H_3TpFPC(-S-PEG(7)-OMe)$_3$ in MeOH.

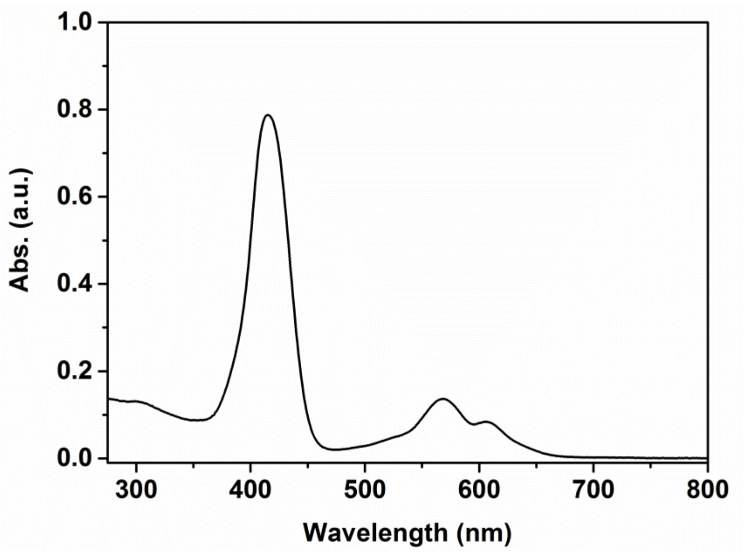

Figure S5: UV-vis spectrum of (2,3,5,6-tetrafluoro-4-(MeO-PEG(7)thiophenyl)corrole H_3TpFPC(-S-PEG(7)-OMe)$_3$ in acetonitrile.

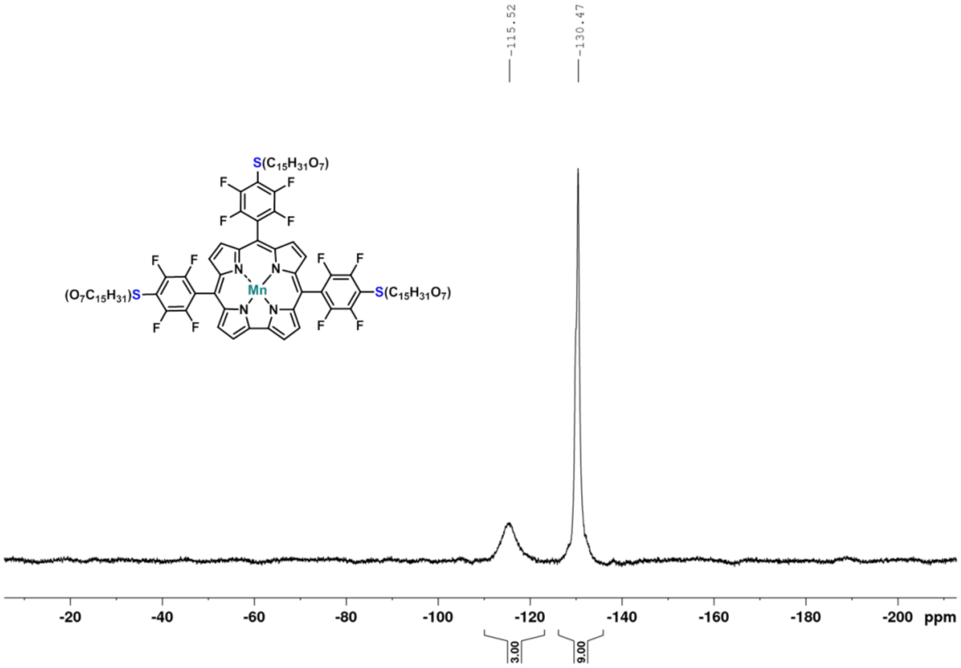

Figure S6: ^{19}F-NMR of Mn (TpFPC)(-S-PEG(7)-OMe)$_3$ in $CDCl_3$.

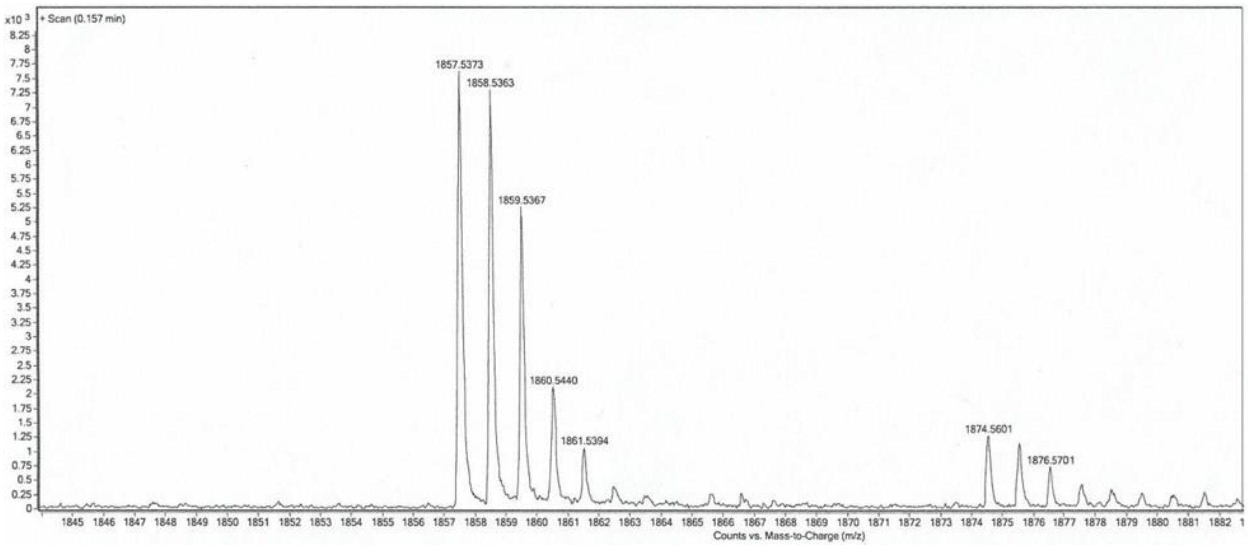

Figure S7: HRMS of Mn (TpFPC)(-S-PEG(7)-OMe)$_3$ in MeOH.

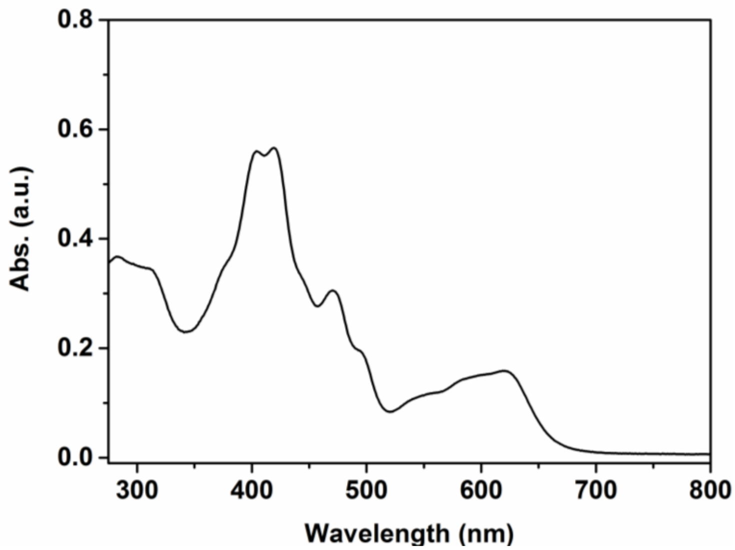

Figure S8: UV-vis of Mn (TpFPC)(-S-PEG(7)-OMe)$_3$ in ACN.

Characterization of Mn-Corrole on Carbon Paper electrode

XPS measurements before and after electrocatalysis

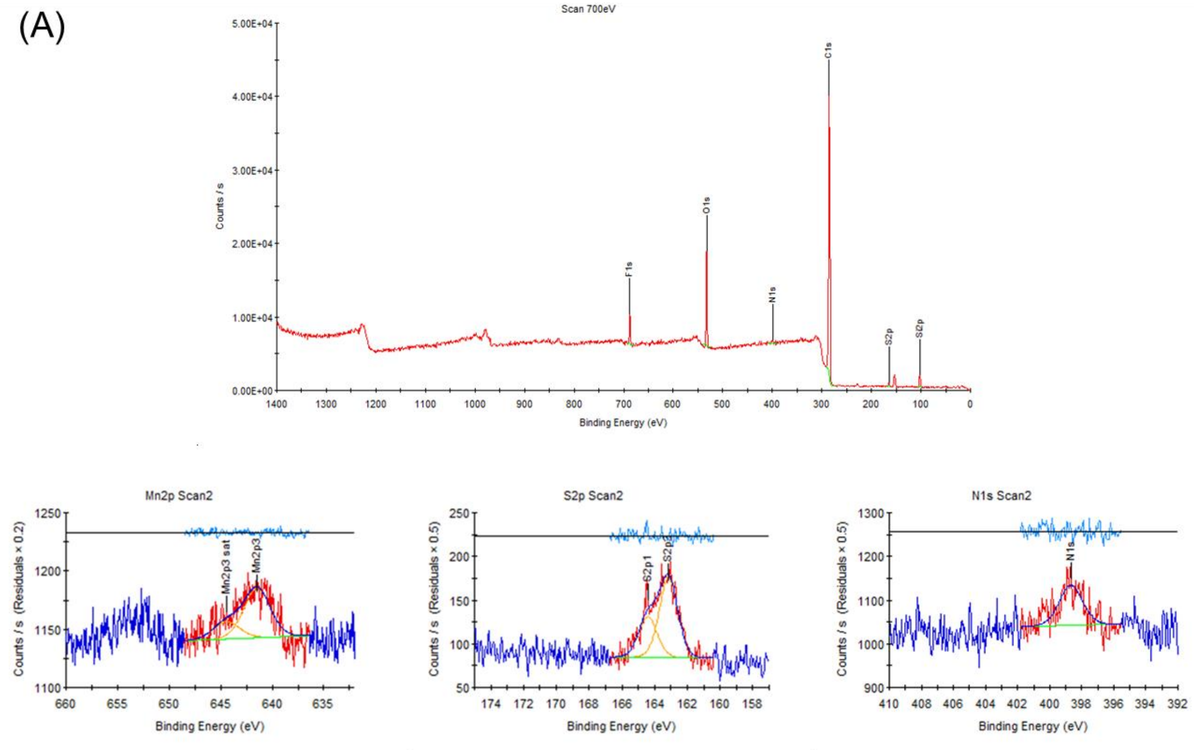

(B)

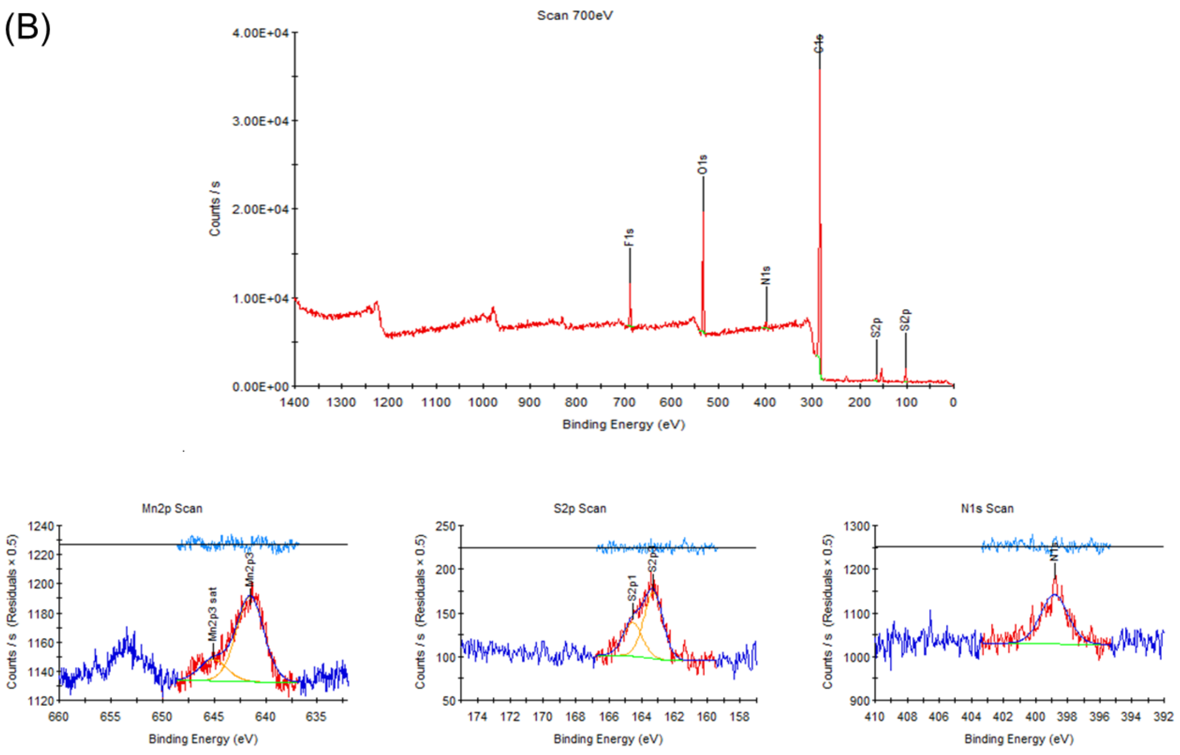

Figure S9: (A) High resolution XPS spectra of the Mn-Corrole on Carbon Paper electrode before and **(B)** after electrocatalysis reaction at -1.25 V vs Ag/AgCl over 5 h CPE, corresponding Mn2p, S2p and N1s, binding energy regions and the peak quantification of Mn to N (ratios Mn/S = 1/3, Mn/N = 1/4) in each case, indicating that the manganese ion is located in the center of the corrole macrocycle and Mn-Corrole is adsorbed on the electrode surface in an unmodified way before as well as after electrocatalysis.

Electrochemical measurements

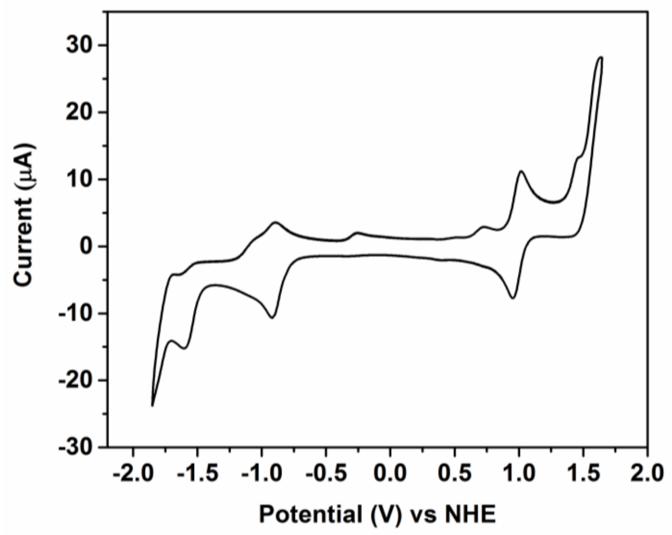

Figure S10: Cyclic voltammetry of Mn-Corrole dissolved in CH_3CN under Ar containing 0.1 M $TBAPF_6$ as supporting electrolyte with glassy carbon as working, platinum wire as counter and nonaqueous pseudo-Ag/AgCl as reference electrode with a scan rate of 100 mV s^{-1}.

Electro paramagnetic resoncance (EPR) measurement

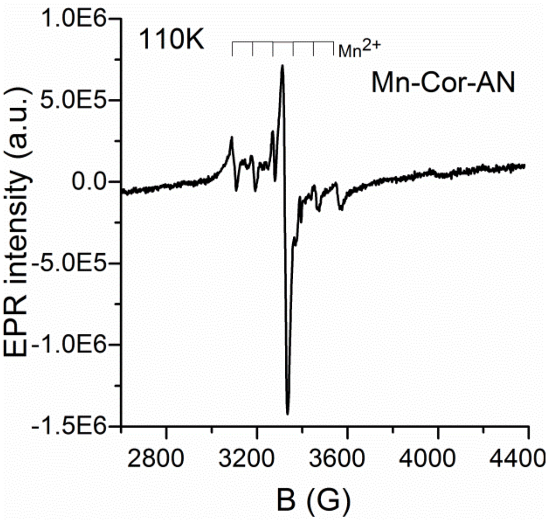

Figure S11: EPR spectrum of Mn-Corrole in ACN under argon after electrochemical reduction, indicating the formation of a Mn(II)species.

Heterogeneous electrochemistry with Mn-Corrole-Carbon Paper

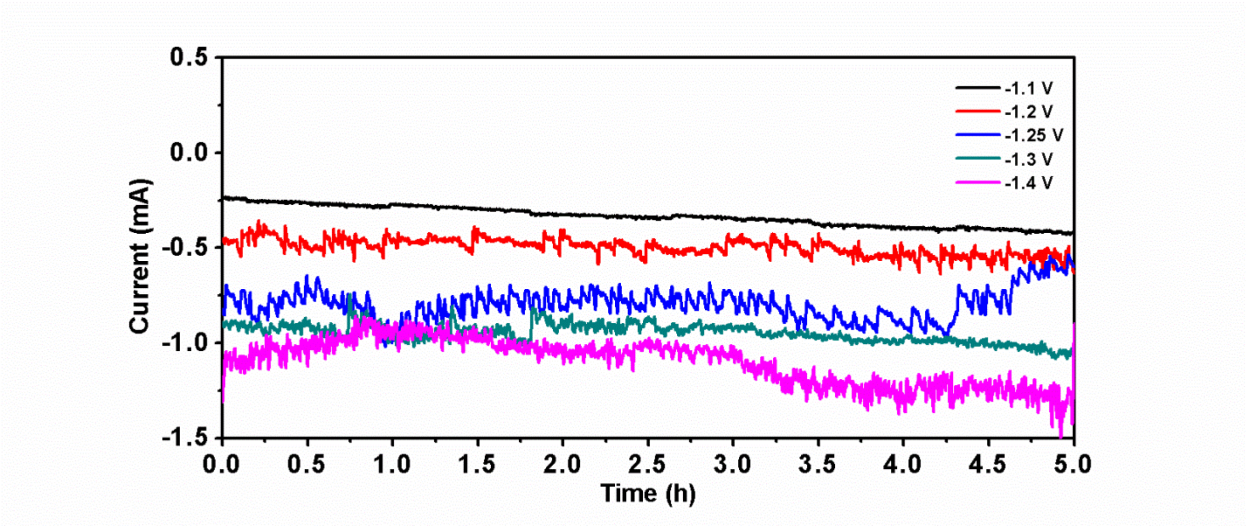

Figure S12: Controlled potential electrolysis of the electrochemical CO_2 reduction at varying potentials (-1.1 to -1.4 V vs Ag/AgCl) with Mn-Corrole-CP over 5 h, pH 6.

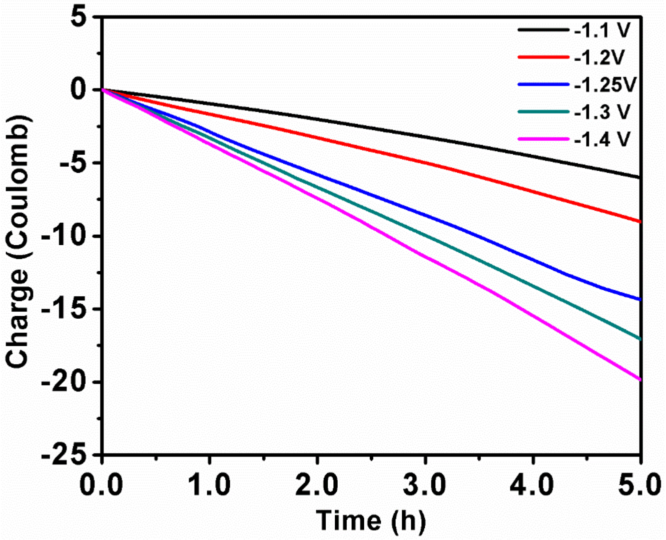

Figure S13: Charge passed vs time plot for the controlled potential electrolysis over 5 h at varying potentials with the Mn-Corrole modified-CP electrode.

(A)

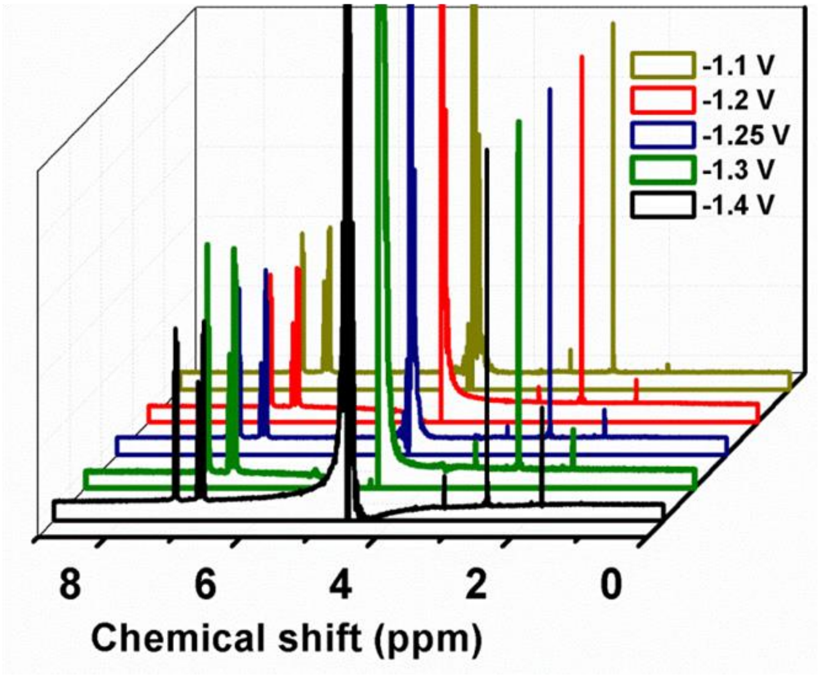
-1.1 V
-1.2 V
-1.25 V
-1.3 V
-1.4 V
8
6
4
2
0
Chemical shift (ppm)

(B)

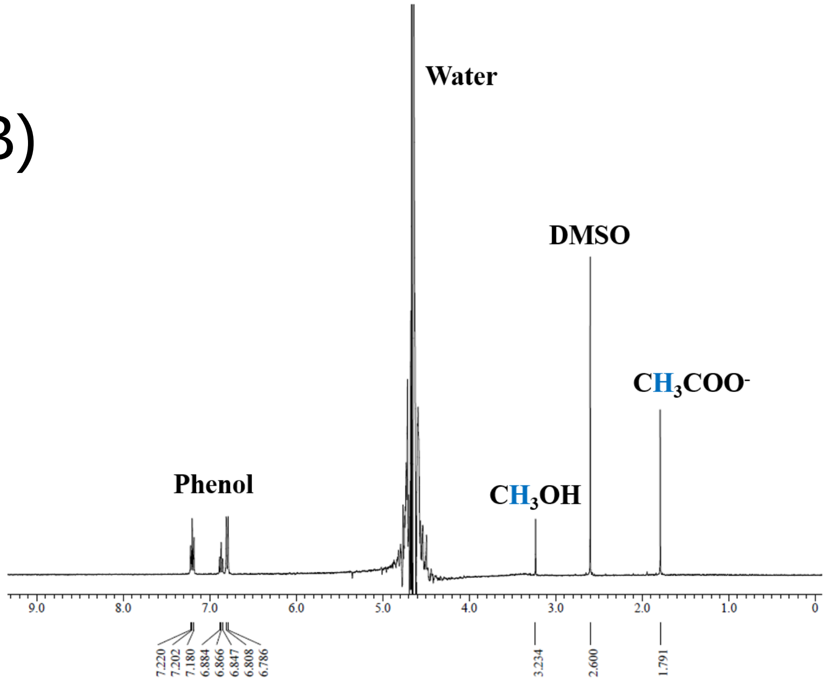
Water
DMSO
CH3COO-
Phenol
CH3OH
9.0
8.0
7.0
6.0
5.0
4.0
3.0
2.0
1.0
0
7.220
7.202
7.180
6.884
6.866
6.847
6.808
6.786
3.234
2.600
1.791

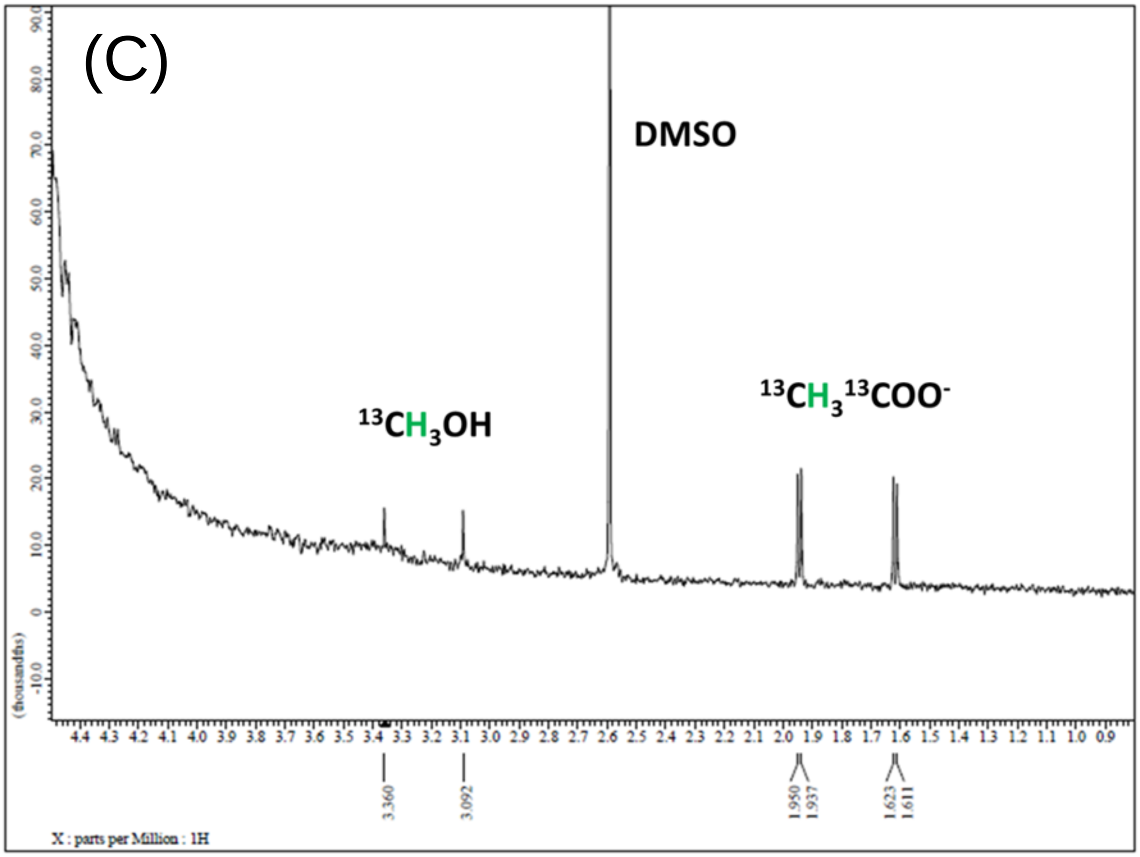

Figure S14: A) ^{1}H-NMR spectra of the liquid products formed after 5 h of constant potential electrolysis at different potentials by Mn-Corrole-CP electrode (-1.1 V to -1.4 V vs Ag/AgCl, 0.1 M phosphate buffer, pH 6, phenol as internal standard). B) ^{1}H-NMR spectrum of the liquid products formed after 5 h of controlled potential electrolysis at -1.25 V vs Ag/AgCl by Mn-Corrole-CP electrode (0.1 M phosphate buffer, pH 6, phenol as internal standard). C) ^{1}H-NMR spectrum of the liquid products formed after $^{13}CO_2$ reduction at -1.25 V vs Ag/AgCl for 5 h by Mn-Corrole modified Carbon Paper electrode (pH 6, 0.1 M phosphate buffer).

Calculation of Faradaic efficiency and Turn over frequency

Faradaic efficiencies (FE%) of the obtained products were calculated with the following equation;[12]

$$\mathrm{FE\%} = \frac{\text{amount of product} \cdot n \cdot F}{Q} \cdot 100$$

where

n = number of electrons involved in formation of one product molecule

F = Faradaic constant (96485 C mol^{-1})

Q = amount of charge passed through the working electrode (C)

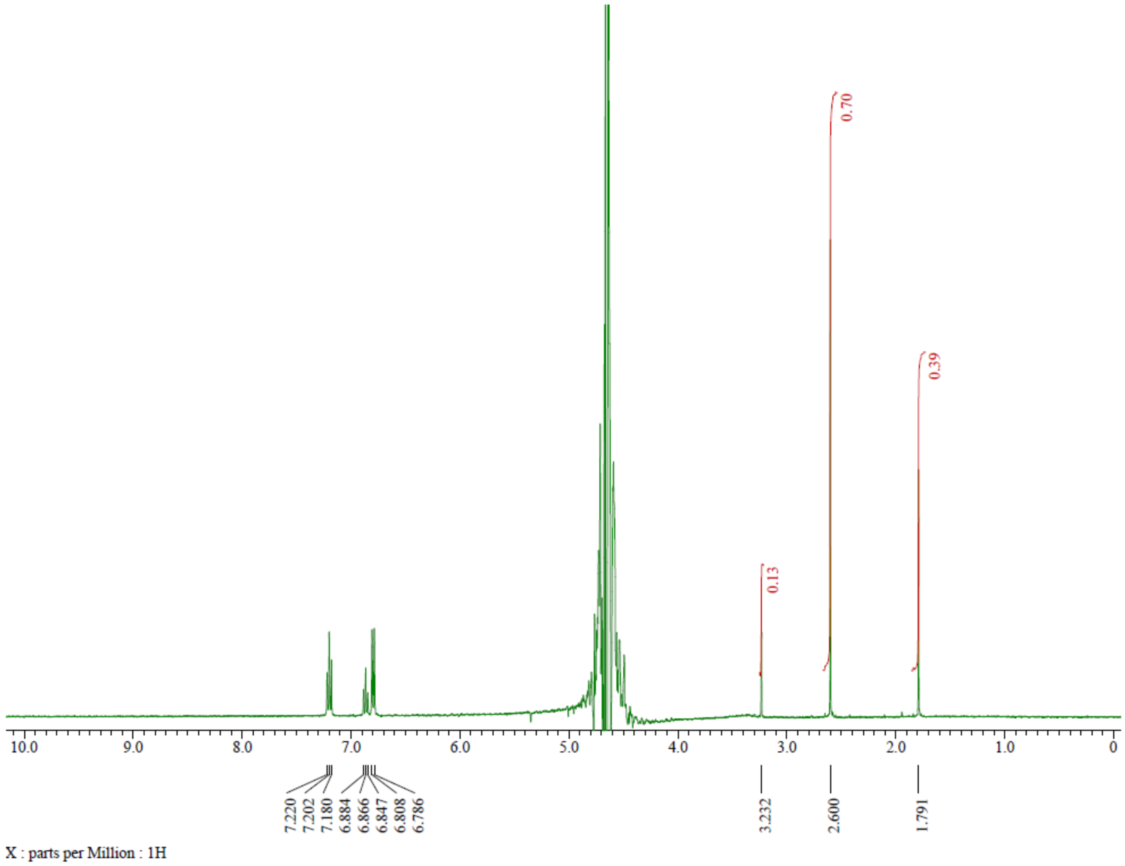

Figure S15: ^{1}H-NMR spectrum of the liquid products formed after CO_2 reduction at -1.25 V vs Ag/AgCl for 5 h by Mn-Corrole modified Carbon Paper electrode (pH 6, 0.1 M phosphate buffer).

Molar concentration (mM) of the reduced product was calculated from the ^{1}H-NMR spectrum using the following procedure:
NMR sample contains:

400 μL analyte + 50 μL D_2O + 50 μL external standard (7 mM DMSO + 25 mM phenol)

Total volume of the NMR sample = 500 μL
Effective strength of DMSO in the sample = (7 mM · 50 μL)/500 μL= 0.7 mM
^{1}H-NMR signals of acetate and methanol were integrated and normalized with respect to DMSO.
From the ^{1}H-NMR integration results: amount of acetate in the sample = 0.39 mM
In 400 μL analyte the amount of acetate is = 0.39 mM · 0.4 mL = 0.156 μmol
Total volume of the electrolyte taken for the CPE = 30 mL
So, total amount of acetate produced during electrolysis (at -1.25 V) = 11.7 μmol

Total amount of charge passed after 5 h = 14.4 C
Faradaic efficiency of acetate at -1.25 V after 5 h of electrolysis:

$$FE\% = \frac{11.7 \cdot 10^{-6} \cdot 8 \cdot 96485}{14.4} \cdot 100$$

$$= 62.7$$

Turn Over Frequency (TOF) was calculated using the equation;[12]

$$TOF = \frac{i \cdot FE}{N \cdot F \cdot n_{cat}}$$

$$= 8.25\ h^{-1}$$

where

i = current (A)
FE = Faradaic efficiency for acetate
N = Number of electrons in the half reaction (N = 8 for CO_2 to acetate conversion)
F = Faradaic constant (96485 C mol^{-1})
n_{cat} = total moles of the catalyst employed for the electrolysis

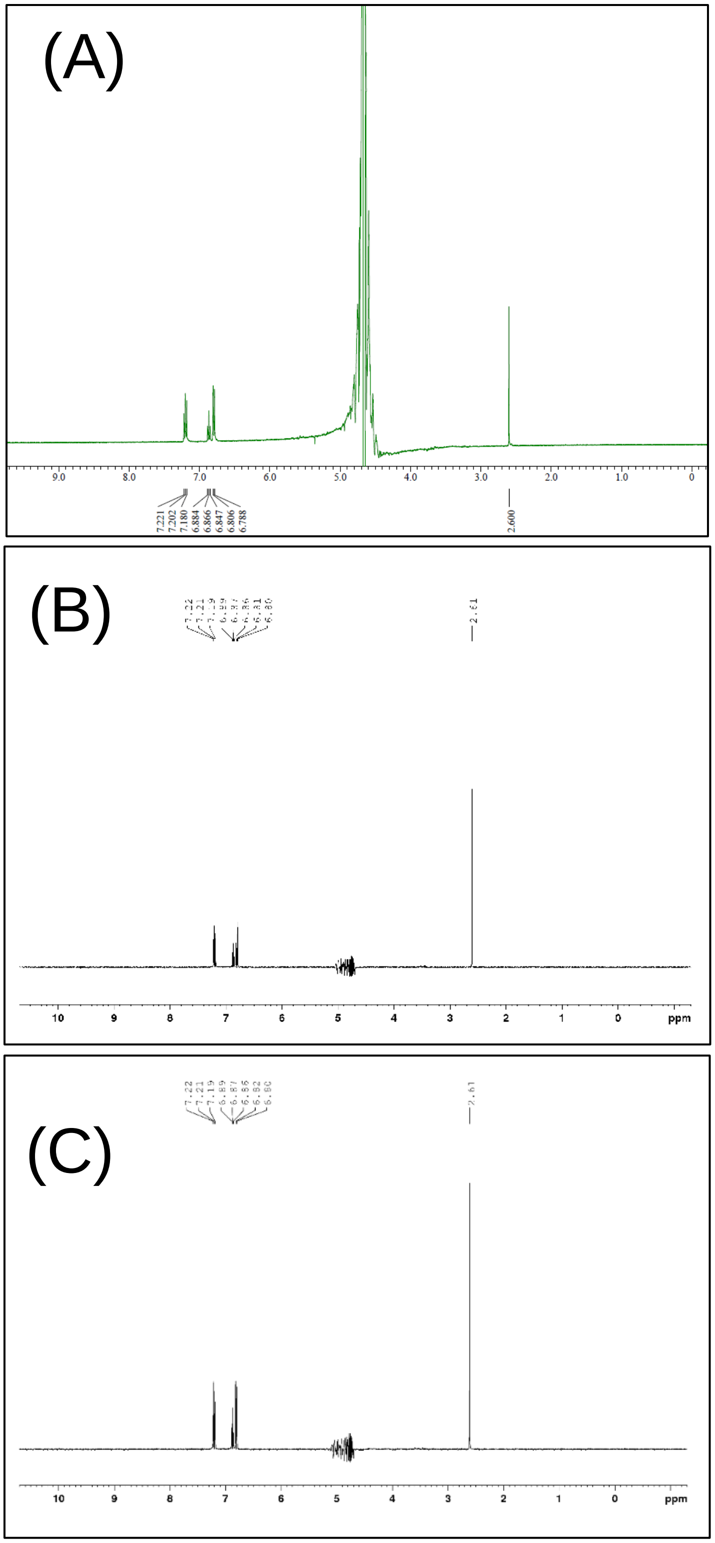

Figure S16: A)^{1}H-NMR spectrum of the electrolyte after 5 h of CO_2 electrolysis with blank CP as working electrode at -1.25 V vs Ag/AgCl (Phenol and DMSO as internal standard). B) ^{1}H-NMR spectrum of the electrolyte after electrocatalysis with free-base corrole-CP at -1.25 V vs Ag/AgCl after 5h CPE. C) ^{1}H NMR spectrum of electrolyte with PEG_7-OMe-SH on CP at -1.25 V vs Ag/AgCl after 5h CPE (20 mL electrolyte).

After catalysis characterization

The following characterizations of the catalyst were performed after the controlled potential experiment to check the stability and molecular integrity of the catalyst. After electrolysis, the catalyst was dissolved of the electrode with acetonitrile, which was used for further analysis. From its electronic absorption spectrum, no shift of the characteristic bands was observed, except a hypochromic shift of the charge transfer band, which could be assigned to the absorption of CO_2 and H_2O on the axial site of the Mn-Corrole (Figure S17A).

The cyclic voltammogram measured after 5 h of CO_2 electrolysis showed no shift of the cyclic voltammetry curve along with nearly identical current density (Figure S17B) imparting stability of the catalyst after extended hours of activity.

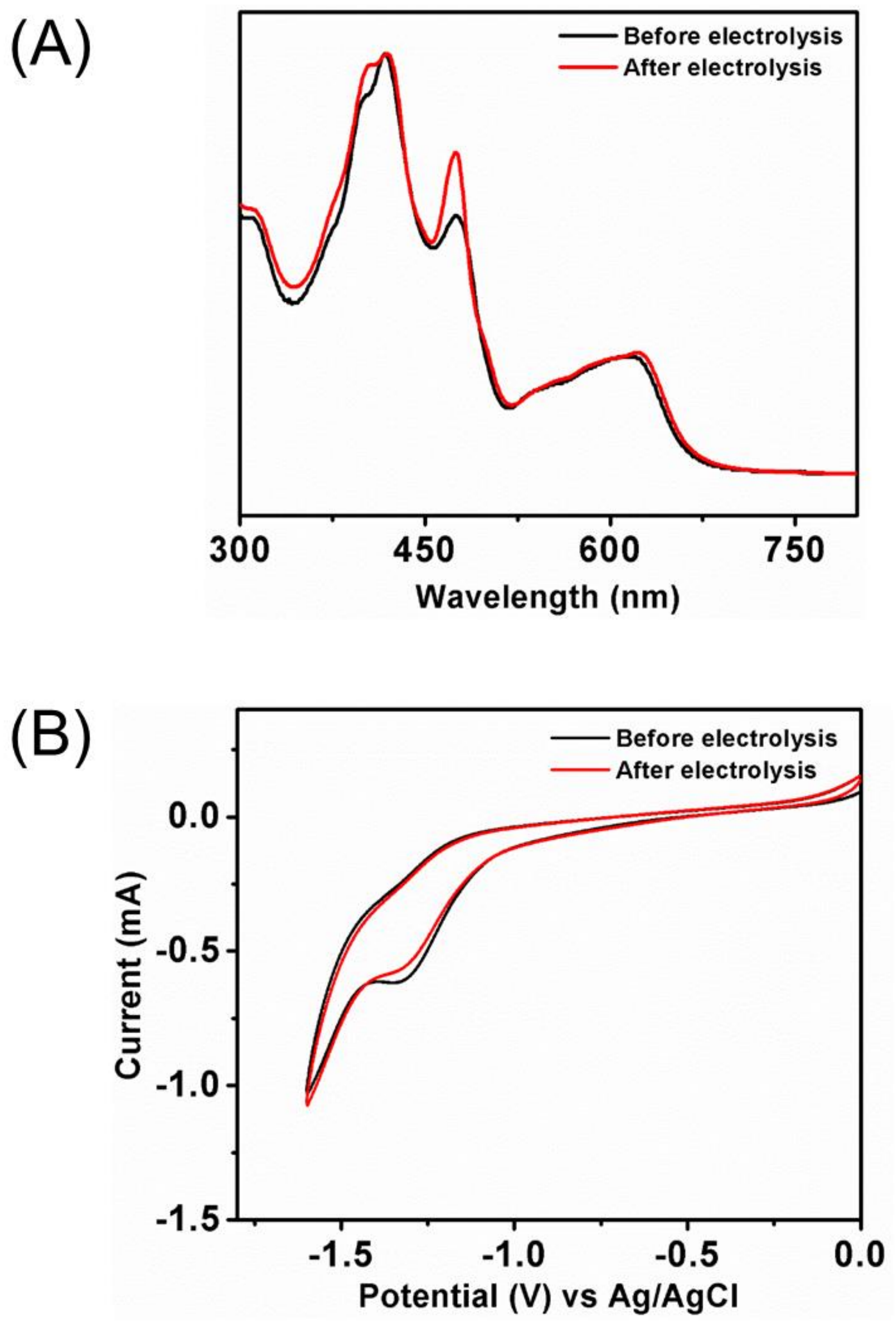

Figure S17: Characterization of the Mn-Corrole after 5 h CPE at -1.25 V vs Ag/AgCl. **(A)** Electronic absorption spectra before and after electrolysis in ACN. **(B)** Electrochemical characterization of the Mn-Corrole-CP electrode by CV: before and after electrolysis.

Effect of Deuterium substitution

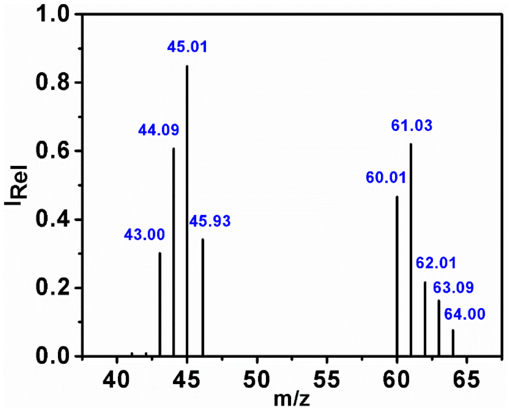

Figure S18: Mass spectrum of deuterated acetic acid obtained after CO_2 electrolysis at -1.25 V vs Ag/AgCl by Mn-Corrole-CP in pH 6 phosphate buffer (D_2O/H_2O = 1/5) for 6000 s.

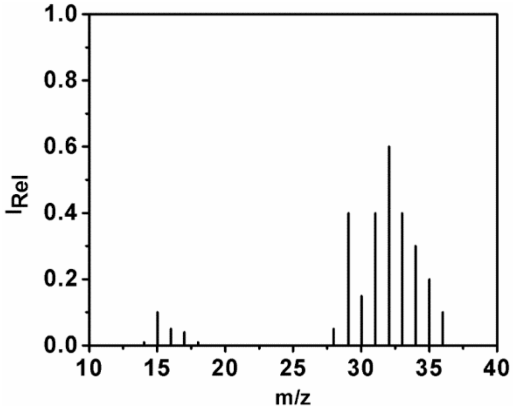

Figure S19: Mass spectrum of deuterated methanol obtained after CO_2 electrolysis at -1.25 V vs Ag/AgCl by Mn-Corrole-CP in pH 6 phosphate buffer (D_2O/H_2O = 1/5) for 6000 s.

Reduction studies of possible intermediates

To get a deeper insight of which intermediates could be involved in the CO_2 reduction mechanism, contant potential electrolysis has been conducted with two different possible intermediates, formic acid and oxalic acid, under the same conditions as used for CO_2 reduction.
10 mM formic acid was added to 30 mL electrolyte (pH 6, 0.1 M phosphate buffer) to perform reduction studies. ^{1}H-NMR analysis of the electrolyte after 6000 s CPE at -1.25 V vs Ag/AgCl, confirmed the presence of methanol due to formic acid reduction as singlet at 3.23 ppm (Figure S20). Apart from methanol no other product was detected from the ^{1}H-NMR and GC-MS after formic acid reduction.
Same kind of reaction was also performed with 10 mM oxalic acid in 0.1 M phosphate buffer, pH 6. After 6000 s of controlled potential electrolysis at -1.25 V vs Ag/AgCl, the ^{1}H-NMR spectrum of the electrolyte showed the formation of acetic acid as singlet at 1.79 ppm (Figure S21). From these results it can be concluded that formic acid and oxalic acid are two key intermediates for the methanol and acetic acid production respectively. The two pathways are different, which was confirmed from the absence of acetic acid during the formic acid reduction and methanol being absent after oxalic acid reduction.

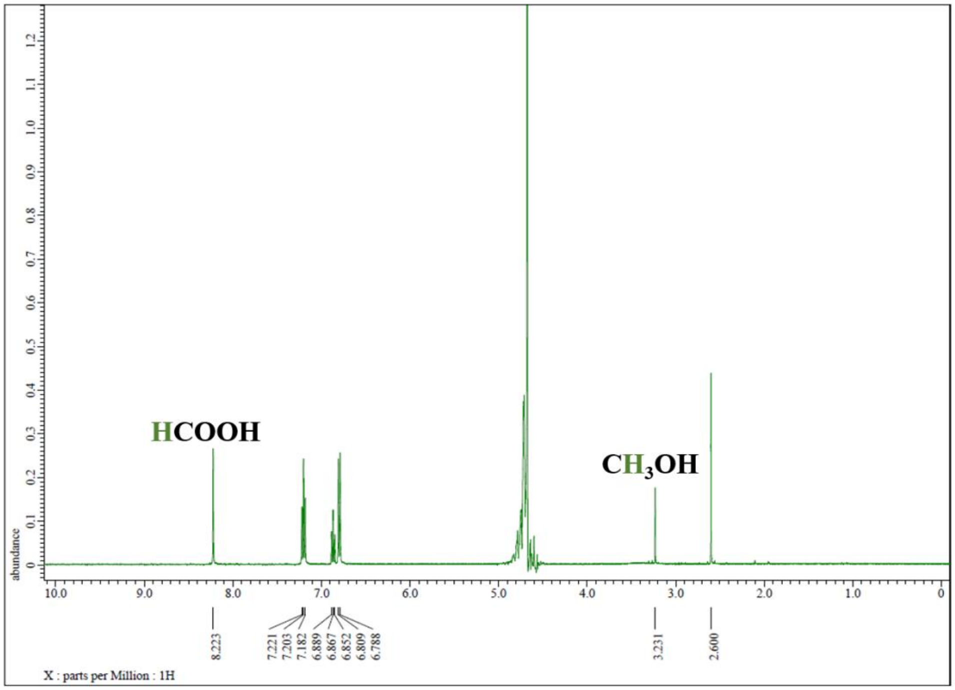

Figure S20: ^{1}H-NMR spectrum of the electrolyte after 10 mM formic acid reduction with Mn-Corrole-CP at -1.25 V vs Ag/AgCl for 6000 s.

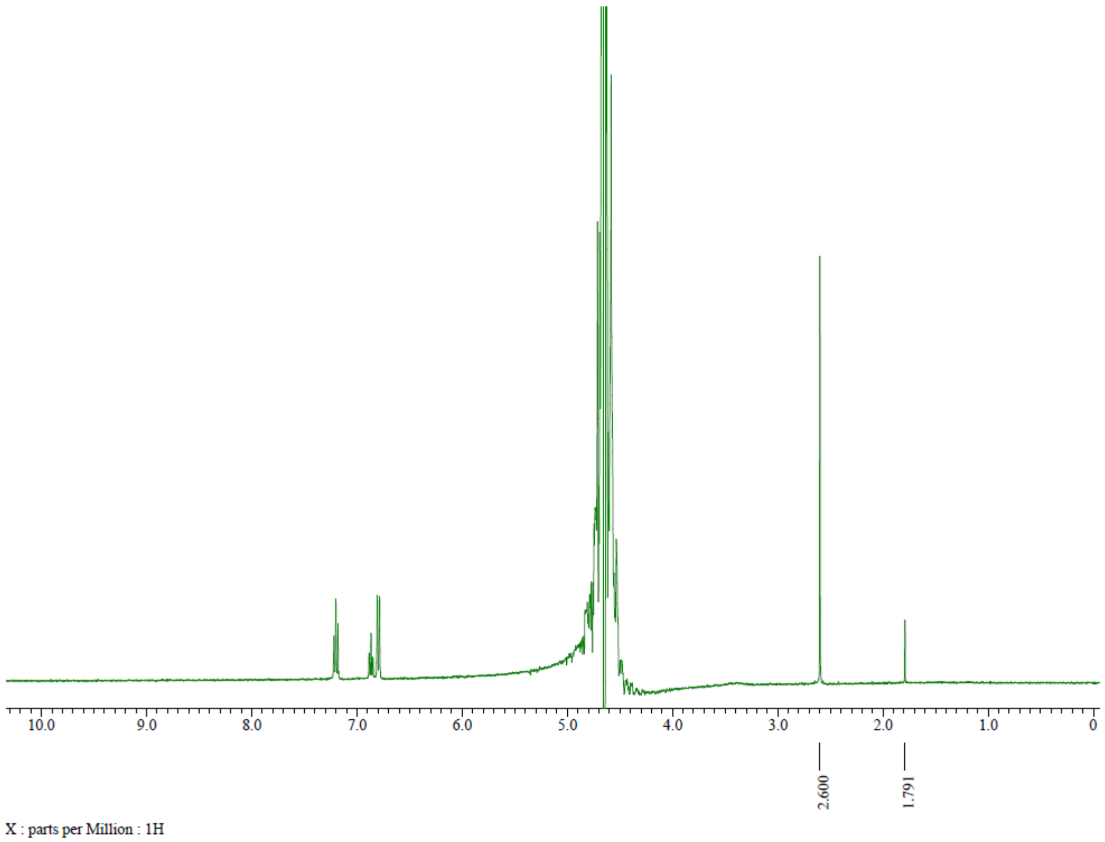

Figure S21: ^{1}H-NMR spectrum of the electrolyte after 10 mM oxalic acid reduction with Mn-Corrole-CP at -1.25 V vs Ag/AgCl for 6000 s.

DFT calculations

The geometry optimization and frequency calculations of all the structures were B3LYP[13] with Grimme's dispersion correction D3[14] as implemented in Gaussian 09 software package[15]. Pople basis set of valence double ζ quality (6-31G*)[16] with polarization function over all the atoms (Mn, S, O, N, C and H). The integral equation formalism of the polarized continuum model (IEF-PCM)[17] was used to consider the solvent water. DFT calculations were done using a Mn-Corrole complex in which the PEG(7)-OMe unit was replaced with PEG(1)-OMe unit (Optimized Mn(III)-Cor structure is give in Figure S25 and the coordinates are given on p.31-33). Analytical vibrational frequencies were computed to verify the nature of the stationary states.

The results obtained were used to calculate the binding energy differences between various geometries for CO_2 reduction. Only Mn-Corrole molecule was modeled and not the support electrode CP since the latter doesn't influence the electroactivity. Theoretical redox potential calculations were carried out using self-consistent reaction field (SCRF) approach based on the integral equation formalism of the polarized continuum model (IEF-PCM) level of theory and the solvation free energies (ΔG_s^o) for the complex in all the oxidation states (Mn(III), Mn) was found out using default options as given in Gaussian 09[18].

Adsorption and subsequent activation of CO_2

On monitoring the change in the Mulliken charges on reduction of the metal center in Mn-Corrole from Mn(III) to Mn(II)[19], it can be concluded that charge on the metal center has changed from +1.129 (Mn(III)) to +1.029 (Mn(II)) while the charges on nitrogen atoms of both the species have an average value of *ca*. -0.76. Further CO_2 molecule gets adsorbed onto the 1 e^- reduced Mn[19] centre *via* its C atom and the geometry of the CO_2 molecule is a bent one (<O–C–O: 145.6°, C–O: 1.19 Å) as opposed to the linear form found in its free state which evidences the presence of a more stable configuration (chemisorption) then other weak adsorptions (physisorption)[20] between CO_2 and the metal center (Figure S22(A)). This configuration is called as bent (δ) CO_2 and this initial adsorption is accompanied by the charge transfer to the CO_2 molecule from the metal center resulting in the formation of metal bound carboxyhydroxyl intermediate (Mn(III)–COOH) (Equation S4) where the bending in the protonated CO_2 molecule is further enhanced (<O–C–O: 120.8°, C–O: 1.20 and 1.35 Å) (Figure S22(B)).

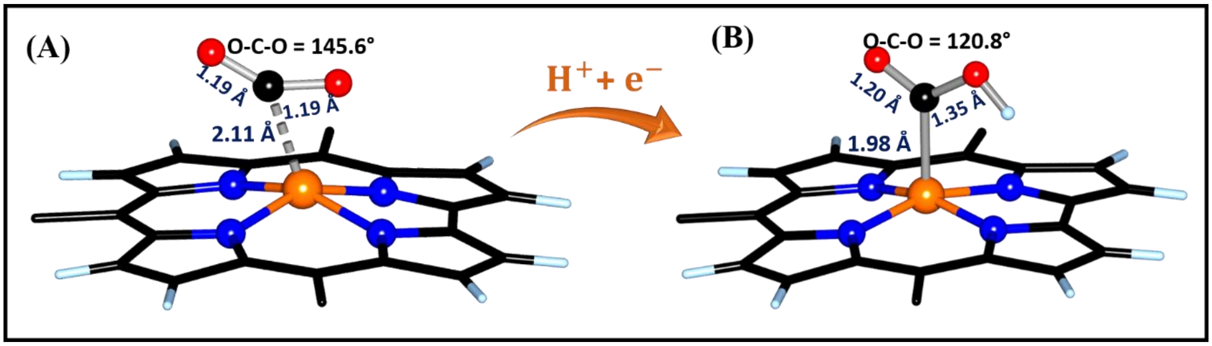

Figure S22: Optimized structures of **(A)** Mn-$CO_{2\ adsorbed}$ and **(B)** Mn(III)-COOH at B3LYP-D3/6-31g* level of theory (For clarity the S-PEG(7)-OMe unit is not shown).

During this process, the charge on the metal centre in (Mn(III)–COOH) changes to +1.03 which is slightly greater than that in free Mn(III)-Cor and the charges on the carbon and oxygen atoms in CO_2 (C = +0.42, O = -0.42 and -0.57) were found to be different from that of the free CO_2 (C = +1.05; O = -0.52 each) implying an occurrence of charge transfer. To probe into the origin of charge transfer and the underlying mechanism, the interaction between Mn site of Mn(III) Corrole and carboxyhydroxyl species on the Mn(III)-COOH intermediate was studied. On examination of HOMO orbital of Mn(III)-COOH (Figure S23), a charge transfer transition between Mn(III) d_{z^2} orbital (35.64%) to π* antibonding orbital (55.59%) of CO_2H was observed.[21]

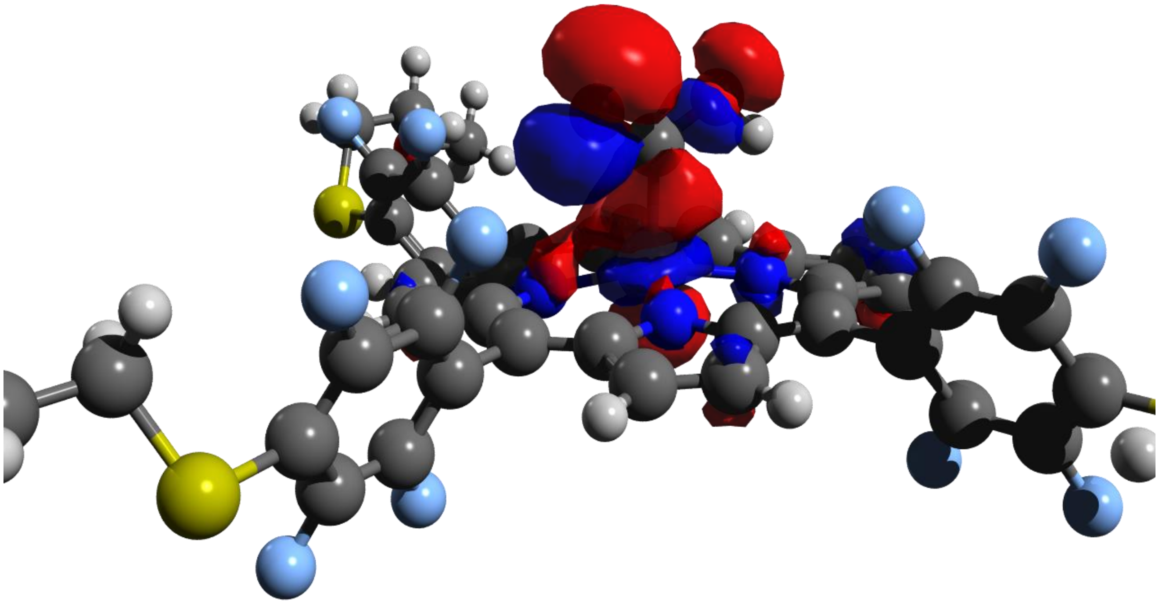

Figure S23: Spatial representation of HOMO orbital of Mn(III)-COOH showing interaction of Mn(III) with coordinated $COOH^{\bullet}$.

Possible equations involved in CO_2 electroreduction along with respective reaction free energies:

$$Mn(III) + e^- \rightarrow [Mn(II)]^- \cdots\cdots (S1)\ \Delta G_{aq} = -2.88\ eV$$

$$alternative\ (Hydride\ formation)$$

$$[Mn(II)]^- + H^+ + e^- \rightarrow [Mn(II) - H]^- \cdots\cdots (S2)\ \Delta G_{aq} = +4.18\ eV$$

$$[Mn(II)]^- + CO_2 \rightarrow [Mn(II) - CO_{2\,ads}]^- \cdots\cdots (S3)\ \Delta G_{aq} = -0.21\ eV$$

$$[Mn(II) - CO_{2\,ads}]^- + H^+ \rightarrow [Mn(III) - COOH]^0 \cdots\cdots (S4)\ \Delta G_{aq} = +0.74\ eV$$

Carbon Monoxide Pathway:

$$[\,Mn(III) - COOH]^0 + H^+ + e^- \rightarrow [Mn(III) - CO]^0 + H_2O \cdots\cdots (S5)$$

Acetate Pathway:

$$[Mn(III) - COOH]^0 + CO_2 + e^- \rightarrow [Mn(III) - OCO - COOH]^- \cdots\cdots (S6)\ \Delta G_{aq} = -1.04\ eV$$

$$[Mn(III) - OCO - COOH]^- + 2H^+ + 2e^- \rightarrow [Mn(III) - OCO - CHO]^0 + H_2O \cdots (S7)\ \Delta G_{aq} = -0.77\ eV$$

$$[Mn(III) - OCO - CHO]^0 + 2H^+ + 2e^- \rightarrow [Mn(III) - OCO - CH_2OH]^0 \cdots\cdots (S8)\ \Delta G_{aq} = -2.40\ eV$$

$$[Mn(III) - OCO - CH_2OH]^0 + 2H^+ + 2e^- \rightarrow [Mn(III) - OCO - CH_3]^- + H_2O \cdot\cdot (S9)$$

Methanol pathway:

$$[Mn(III) - COOH]^0 + H^+ + e^- \rightarrow [Mn(III) - HCOOH]^0 \cdots\cdots (S10)$$

$$[Mn(III) - HCOOH]^0 + e^- \rightarrow [Mn(III) - HCO^{\cdot}]^0 + OH^- \cdots\cdots (S11)$$

$$Mn(III) - HCO^{\cdot} + e^- + H^+ \rightarrow Mn(III) - HCHO \cdots\cdots (S12)$$

$$Mn(III) - HCHO + e^- + H^+ \rightarrow Mn(III) - OCH_3 \cdots\cdots (S13)$$

$$Mn(III) - OCH_3 + e^- + H^+ \rightarrow Mn(III) + CH_3OH \cdots\cdots (S14)$$

Computational Redox Potential

The half-cell reaction corresponding to one electron reduction can be given as

Ox^{n} (solv) + e^{-} → Red^{n-1} (solv)

where Ox = oxidized species and Red = reduced species.

Now the corresponding thermodynamic cycle for Gibbs free energy calculation is given as follows (Figure S24)

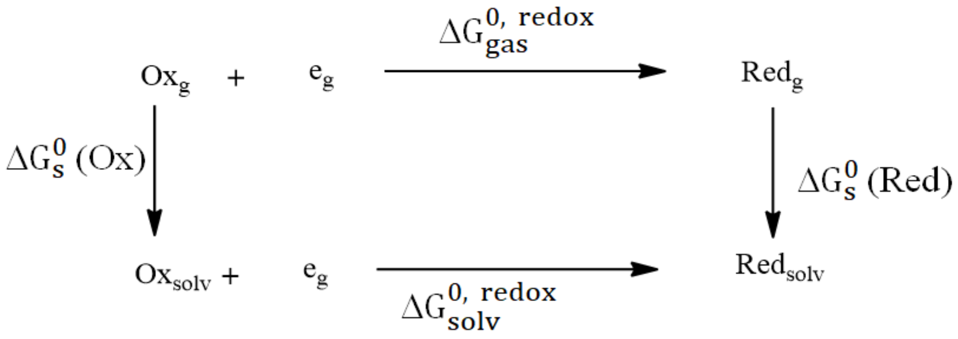

Figure S24: Thermodynamic cycle for the calculation of Gibbs free energy for the one electron reduction process.

$\Delta G^{0,redox}_{solv}$, which is the standard Gibbs free energy of a redox half reaction in a solution which can be calculated *via* the following equation:

$\Delta G^{0,redox}_{solv} = \Delta G^{0,redox}_{gas} + \Delta G^{0}_{s}(Red) - \Delta G^{0}_{s}(Ox)$

Where $\Delta G^{0,redox}_{gas}$, is the free energy change in the gas phase, $\Delta G^{0}_{s}(Red)$ and $\Delta G^{0}_{s}(Ox)$, are the solvation free energies of the reduced and oxidized forms respectively.

Further $\Delta G^{0,redox}_{solv} = -F \times E^{0}_{calc}$;

where F = 23.06 kcal mol^{-1} V^{-1} and E^{0}_{calc} = standard one electron reduction potential.

E^{0}_{calc} Vs (Ag/AgCl) = $E^{0}_{calc} - E^{0}_{SHE} - E_{exp,Ag/AgCl}$

Where E^{0}_{SHE} = absolute potential of the standard hydrogen electrode, in acetonitrile it is reported to be at 4.429 V[22] and $E_{exp,Ag/AgCl}$= experimental redox potential of Ag/AgCl couple which is 0.222 V relative to the standard hydrogen electrode.

Absolute potential for the standard hydrogen electrode in water, E^{0}_{SHE} = 4.281 V

For one electron reduction of Mn-corrole^{0} → Mn-corrole^{1-}

$E^{0,\ Mn(III)\rightarrow Mn(II)}_{calc}$= +3.356 V

$E^{Mn(III)\rightarrow Mn(II)}_{calc}$ Vs $(Ag/AgCl)_{acetonitrile}$ = -1.29 V (Experimental value : -1.14 V)

$E^{Mn(III)\rightarrow Mn(II)}_{calc}$ Vs $(Ag/AgCl)_{water}$ = -1.15 V (Experimental value : -1.20 V)

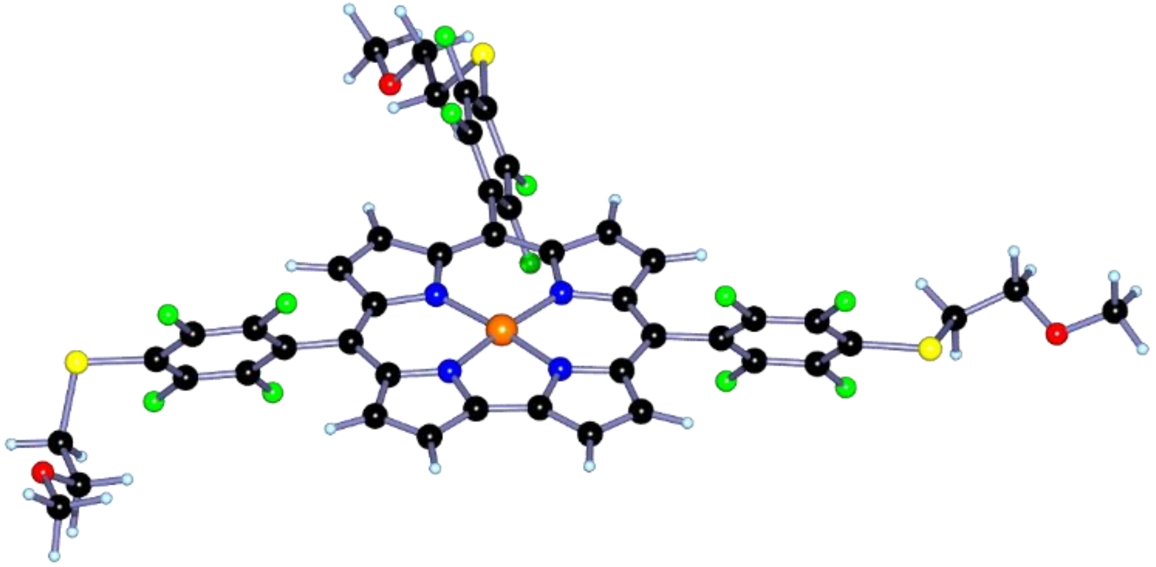

Figure S25: Optimized structure of Mn(III)-Corrole at B3LYP-D3/6-31g* level of theory (orange = manganese, blue = nitrogen, green = fluorine, orange = phosphorous, red = oxygen, black = carbon, pale blue = hydrogen).

Coordinates of optimized geometry of Mn(III)-Corrole

Mn	-0.14197	-1.43865	0.09911
N	-1.47836	-2.72606	0.01715
N	-1.42970	-0.06900	0.23323
N	1.34654	-0.28367	0.14566
N	0.97396	-2.91651	-0.04555
C	-1.05905	-4.05530	-0.12036
C	-2.20355	-4.87428	-0.19220
C	-3.31245	-4.03106	-0.10038
C	-2.84371	-2.69095	0.03024
C	-3.51970	-1.44979	0.14054
C	-2.83508	-0.23012	0.23811
C	-3.42590	1.06540	0.36325
C	-2.41575	1.99057	0.42265
C	-1.17059	1.28943	0.34548
C	0.12021	1.84743	0.37523
C	1.30637	1.09542	0.28850
C	2.64703	1.59101	0.35167
C	3.49795	0.52133	0.23983
C	2.70892	-0.66212	0.10403
C	3.19212	-1.97201	-0.02924
C	2.32715	-3.09188	-0.11067
C	2.57595	-4.48691	-0.27044
C	1.34723	-5.14858	-0.29839

C	0.34824	-4.16415	-0.15964
C	-5.00772	-1.45484	0.14584
C	4.65994	-2.20796	-0.08348
C	0.24087	3.32789	0.50632
C	0.73666	4.12126	-0.53483
C	0.86730	5.50056	-0.40990
C	0.47182	6.17201	0.75313
C	-0.02941	5.38392	1.79602
C	-0.13110	3.99985	1.67504
F	1.11893	3.54975	-1.68735
F	1.37600	6.18709	-1.44617
S	0.66106	7.92721	0.96745
F	-0.41582	5.94952	2.94694
C	-5.75909	-0.86473	-0.87787
C	-7.14965	-0.87061	-0.86433
C	-7.87605	-1.50622	0.15089
C	-7.12735	-2.10665	1.17093
C	-5.73481	-2.07314	1.16868
F	-5.13799	-0.25742	-1.90149
F	-7.79335	-0.25583	-1.87244
S	-9.65357	-1.48608	0.24147
F	-7.74108	-2.72289	2.18871
F	-5.08707	-2.66417	2.18527
C	5.45890	-1.71377	-1.12159
C	6.82675	-1.96059	-1.17726
C	7.48488	-2.69621	-0.18492
C	6.68774	-3.19999	0.85025
C	5.31720	-2.96249	0.89549
F	4.90433	-0.99976	-2.11386
F	7.51671	-1.47758	-2.22555
S	9.21762	-3.08816	-0.23295
F	7.24026	-3.92177	1.83558
F	4.62335	-3.46370	1.93022
H	3.55042	-4.94824	-0.36037
H	1.19113	-6.21336	-0.40998
H	-2.21921	-5.95072	-0.30001
H	-4.34979	-4.33714	-0.12646
H	4.57820	0.55028	0.26220
H	2.92485	2.62781	0.48078
H	-4.48718	1.26545	0.40796
H	-2.52631	3.06252	0.50887
F	-0.61464	3.30793	2.71858
C	-10.12999	-2.20848	-1.38789
C	-9.91386	-3.70931	-1.50714
H	-9.60820	-1.67459	-2.18385
H	-11.19892	-1.98790	-1.47020

O	-10.79118	-4.37021	-0.62547
H	-8.86369	-3.96843	-1.28926
H	-10.11304	-4.01063	-2.55234
C	-10.60687	-5.76989	-0.60927
H	-10.78778	-6.21812	-1.59989
H	-11.32750	-6.17947	0.10275
H	-9.58969	-6.04231	-0.28544
C	9.96864	-1.43915	-0.61969
C	11.36470	-1.38255	-0.02246
H	9.34443	-0.65779	-0.17908
H	10.00845	-1.30015	-1.70135
O	12.13321	-2.41918	-0.59522
H	11.31810	-1.49073	1.07334
H	11.81040	-0.39639	-0.24488
C	13.43170	-2.51403	-0.04499
H	14.01095	-1.59207	-0.21287
H	13.93204	-3.34522	-0.54739
H	13.39993	-2.71422	1.03737
C	-0.31186	8.57470	-0.46252
C	-0.37427	10.09531	-0.35853
H	-1.32168	8.15813	-0.42723
H	0.16601	8.28408	-1.39916
O	-1.10421	10.55412	-1.47807
H	0.64260	10.52357	-0.35381
H	-0.86578	10.39851	0.58093
C	-1.23775	11.96088	-1.50222
H	-1.77333	12.33457	-0.61491
H	-1.81265	12.21220	-2.39698
H	-0.25794	12.46209	-1.55292

References

[1] K. P. Kuhl, E. R. Cave, D. N. Abram, T. F. Jaramillo, *Energy Environ. Sci.* **2012**, *5*, 7050.
[2] C. Genovese, M. E. Schuster, E. K. Gibson, D. Gianolio, V. Posligua, R. Grau-Crespo, G. Cibin, P. P. Wells, D. Garai, V. Solokha et al., *Nat. commun.* **2018**, *9*, 935.
[3] T. Kim, A. Kargar, Y. Luo, R. Mohammed, E. Martinez-Loran, A. Ganapathi, P. Shah, D. P. Fenning, *ACS Appl. Energy Mater.* **2018**, *1*, 1965.
[4] Y. Liu, S. Chen, X. Quan, H. Yu, *J. Am. Chem. Soc.* **2015**, *137*, 11631.
[5] T. T. H. Hoang, S. Verma, S. Ma, T. T. Fister, J. Timoshenko, A. I. Frenkel, P. J. A. Kenis, A. A. Gewirth, *J. Am. Chem. Soc.* **2018**, *140*, 5791.
[6] Y. Wang, D. Wang, C. J. Dares, S. L. Marquard, M. V. Sheridan, T. J. Meyer, *Proc. Natl. Acad. Sci. USA* **2018**, *115*, 278.
[7] C. Genovese, C. Ampelli, S. Perathoner, G. Centi, *Green Chem.* **2017**, *19*, 2406.
[8] D. T. Gryko, B. Koszarna, *Org. Biomol. Chem.* **2003**, *1*, 350.
[9] S. Gonglach, S. Paul, M. Haas, F. Pillwein, S. S. Sreejith, S. Barman, R. De, S. Müllegger, P. Gerschel, U.-P. Apfel, H. Coskun, A. Aljabour, P. Stadler, W. Schöfberger, S. Roy, *Nat. commun.* **2019**, *10*, 3864.
[10] V. V. Pavlishchuk, A. W. Addison, *Inorg. Chim. Acta* **2000**, *298*, 97.
[11] M. Krejčik, M. Daněk, F. Hartl, *J. Electroanal. Chem.* **1991**, *317*, 179.
[12] S. Lin, C. S. Diercks, Y.-B. Zhang, N. Kornienko, E. M. Nichols, Y. Zhao, A. R. Paris, D. Kim, P. Yang, O. M. Yaghi et al., *Science* **2015**, *349*, 1208.
[13] a) Lee, Yang, Parr, *Phys. Rev. B* **1988**, *37*, 785; b) A. D. Becke, *J. Chem. Phys.* **1993**, *98*, 1372.
[14] S. Grimme, J. Antony, S. Ehrlich, H. Krieg, *J. Chem. Phys.* **2010**, *132*, 154104.
[15] G. W. T. M. J. Frisch, H. B. Schlegel, G. E. Scuseria, M. A. Robb, J. R. Cheeseman, G. Scalmani, V. Barone, B. Mennucci, G. A. Petersson, H. Nakatsuji, M. Caricato, X. Li, H. P. Hratchian, A. F. Izmaylov, J. Bloino, G. Zheng, J. L. Sonnenberg, M. Hada, M. Ehara, K. Toyota, R. Fukuda, J. Hasegawa, M. Ishida, T. Nakajima, Y. Honda, O. Kitao, H. Nakai, T. Vreven, J. A. Montgomery, Jr., J. E. Peralta, F. Ogliaro, M. Bearpark, J. J. Heyd, E. Brothers, K. N. Kudin, V. N. Staroverov, R. Kobayashi, J. Normand, K. Raghavachari, A. Rendell, J. C. Burant, S. S. Iyengar, J. Tomasi, M. Cossi, N. Rega, J. M. Millam, M. Klene, J. E. Knox, J. B. Cross, V. Bakken, C. Adamo, J. Jaramillo, R. Gomperts, R. E. Stratmann, O. Yazyev, A. J. Austin, R. Cammi, C. Pomelli, J. W. Ochterski, R. L. Martin, K. Morokuma, V. G. Zakrzewski, G. A. Voth, P. Salvador, J. J. Dannenberg, S. Dapprich, A. D. Daniels, O. Farkas, J. B. Foresman, J. V. Ortiz, J. Cioslowski, D. J. Fox (Ed.) *Gaussian, Inc.* Revision D, Wallingford CT, **2013**.
[16] W. J. Hehre, R. Ditchfield, J. A. Pople, *J. Chem. Phys.* **1972**, *56*, 2257.
[17] E. Cancès, B. Mennucci, J. Tomasi, *J. Chem. Phys.* **1997**, *107*, 3032.
[18] M. Cossi, G. Scalmani, N. Rega, V. Barone, *J. Chem. Phys.* **2002**, *117*, 43.
[19] K. Sudhakar, A. Mizrahi, M. Kosa, N. Fridman, B. Tumanskii, M. Saphier, Z. Gross, *Angew. Chem. Int. Ed.* **2017**, *56*, 9837.
[20] Y. Toda, H. Hirayama, N. Kuganathan, A. Torrisi, P. V. Sushko, H. Hosono, *Nat. commun.* **2013**, *4*, 2378.
[21] a) I. M. B. Nielsen, K. Leung, *J. Phys. Chem. A* **2010**, *114*, 10166; b) J. Shen, M. J. Kolb, A. J. Göttle, M. T. M. Koper, *J. Phys. Chem. C* **2016**, *120*, 15714.
[22] A. A. Isse, A. Gennaro, *J. Phys. Chem. B* **2010**, *114*, 7894.
